# Supplementary material for: GWAS meta-analysis of psoriasis identifies new susceptibility alleles impacting disease mechanisms and therapeutic targets
Source: Nat Commun. 2025 Feb 28;16:2051. doi: 10.1038/s41467-025-56719-8 (PMC11871359; doi:10.1038/s41467-025-56719-8)

Supplementary material
for

GWAS meta-analysis of psoriasis identifies new susceptibility alleles impacting disease mechanisms and therapeutic targets

Supplementary Methods

Contributing GWAS studies

The 18 contributing studies included 15 datasets with psoriasis cases ascertained via dermatologists or other secondary care specialist physicians (five previously analysed[1]: CASP, ExomeChip, Genizon, PsA and WTCCC2; ten newly genotyped: BSTOP, Erlangen, Estonia, Kiel, Manchester, Michigan, Newfoundland, Scotland, Toronto and UCSF) and three datasets derived from biobanks (Estonian Biobank, HUNT and UK Biobank). Full details of ethics approval, ascertainment, genotyping, quality control, genome-wide imputation, and association testing for the 18 contributing studies are provided separately in Supplementary Data 23.

The newly reported datasets were prepared and analysed by contributing analysis centres using a variety of established software applications. For genotype calling: GenomeStudio v.2011.1[2] and Affymetrix Power Tools v 1.18.0[3]. For quality control: PLINK v1.9[4], KING v1.4 and v1.9[5], BAFRegress v0.9.3[6] and flashpca v2.0[7]. For phasing and genome-wide imputation: EAGLE v2.3[8], Minimac3[9], Beagle v.28Sep18.793[10, 11], PBWT[12] and IMPUTE2[13]. For association testing: PLINK v2.0[4], SNPTEST v2.5.2 or v2.5.4-beta3[14], or SAIGE v0.29[15].

Identification of cross-dataset related samples

Prior to association testing, inter-dataset duplicated and first- or second-degree related participants were identified using KING (version 2.0)[5] by sharing subsets of between 2,502 and 6,864 genotyped markers outside known psoriasis-associated regions. To establish that these numbers of markers should be sufficient for robust identification of duplicates and close relatives, we performed testing on the BSTOP dataset. We considered intra-dataset duplicates, first- and second-degree relationships identified with a maximal set of 73,223 LD-independent markers outside previously reported psoriasis loci and regions of long-range LD to represent the ground truth (i.e., best possible with full genotype data available). We then randomly selected smaller subsets of markers to assess our ability to accurately recover these relationships. We found that under KING's default relationship inference settings, all 10 relationships were recovered down to approximately 2,500 markers, with at most two "false positive" relationships. We therefore specified a target overlap of 5,000 markers between cohorts, albeit due to limited overlap between the arrays used in some studies it was necessary to drop below this target in some cases. Genotype sharing was not possible for any of the three biobank studies, meaning that for these datasets, relationship inference could only be performed against the 15 specialist-ascertained studies.

LD reference panel for statistical fine-mapping

For GCTA-COJO signal identification and fine-mapping we employed a custom LD reference panel comprising genome-wide well-imputed (Mach $r^2 \geq 0.7$) data from six GWAS datasets: CASP, ExomeChip (i.e., including full GWAS content), Genizon, PsA and WTCCC2 from this study, plus previously analysed data from Kiel[16] that was omitted from the current meta-analysis because most samples had been re-genotyped as part of the larger Kiel dataset included here. The reference panel comprised a total of 24,069 samples, and included all variants with an effective sample size N_{eff} > 90% of maximum for the full meta-analysis. To ensure that the reference panel accurately estimates the LD between variants in the meta-analysis study population, and minimises the likelihood of bias in downstream analyses, we calculated the consistency parameter (*s*) defined by the susieR fine-mapping software (v0.12.27).[17, 18] For the 104 loci at which downstream analysis was undertaken, *s* varied from 0.00026 to 0.013 with a mean (median) value of 0.0022 (0.0018). These uniformly small *s* values indicate very good consistency between the association summary statistics of our GWAS meta-analysis and the LD reference panel used for downstream statistical fine-mapping with COJO.

This reference panel was also used to identify a set of 170,786 independent markers for estimation of genomic inflation. These were identified using pairwise LD-pruning in PLINK,[4] using window size 1,500 Kb, step size 150 variants and R^2 threshold 0.2.

Partitioning of genome into distinct LD blocks

We used an optimal procedure[19] to partition the autosomal genome into 1,703 approximately independent LD blocks. The LD reference was based on a curated version of 1000 Genomes Project genotypes[20-22] from which we extracted 503 unrelated samples of European ancestry and excluded variants with minor allele frequency < 0.05. The minimum and maximum allowable number of LD reference variants in each LD block was set to 200 and 10,000, respectively. For each autosome, the maximum number of blocks to consider was chosen to match publicly available results of an older, widely-adopted genome-partitioning method applied to European-ancestry samples.[23, 24] Compared to application of the European-based block boundaries from this older method to our LD reference, we found that block boundaries generated by the optimal method yielded a 600-fold reduction in the cost of splitting autosomes, where cost is defined as the sum of squared correlations between variants from different blocks, restricting to all $r^2 > 0.05$. Chromosome bands corresponding to each LD block were identified using the UCSC Table Browser.[25]

Identification of secondary association signals

For the identification of additional independent association signals within associated regions we used a stringent subset of variants having N_{eff} > 93,252 (90% of maximum possible) and employed GCTA-COJO, version 1.93.3-beta.[26] First, we determined independently associated lead variants using a stepwise model selection procedure (--cojo-slct procedure). For LD blocks with multiple independent signals, we further estimated association statistics for each independent signal conditioned on the other independent signals in the same LD block (--cojo-cond procedure). All analyses used default parameter values (--cojo-p 5e-8, --cojo-

wind 10000, cojo-collinear 0.9, --diff-freq 0.2). We do not report results of this procedure for the MHC block, for two reasons: (i) testing in a subset of datasets for which genotype data were available showed very poor consistency between COJO pseudo-conditional analysis and "gold-standard" genotype-based conditional analysis, likely due to the complex LD structure in the region, suggesting that the COJO-based model selection procedure does not reliably identify independent MHC signals; (ii) a substantial fraction of variants within the MHC region failed to achieve the minimum imputation quality threshold in the HUNT study, leading to few variants having N_{eff} > 93,252 (90% of maximum possible).

Filtering of TWAS associations

For each tissue (blood, sun-exposed skin, sun-unexposed skin), an initial list of TWAS associated genes was identified from S-PrediXcan results based on a Bonferroni-corrected transcriptome-wide significance threshold of 2.18×10^{-6} (22,977 tests performed). In total this comprised 239 gene-tissue pairs. Following the recommendations of Barbeira et al[27], these were further filtered to exclude genes with poor prediction performance (prediction performance p-value > 0.05/239 = 2.09×10^{-4}). Finally, we removed genes where we found evidence of potential LD contamination; that is, evidence suggesting that different causal variants were responsible for psoriasis susceptibility and gene expression differences. To do this, we considered that our earlier analyses demonstrated multiple independent association signals at many psoriasis susceptibility loci, and that eQTL summary statistics may also reflect the effects of multiple regulatory association signals. We downloaded GTEx v7 eQTL summary statistics for each TWAS gene[28] and performed the same COJO procedure as described above to identify independent *cis* eQTL signals, and to generate eQTL association statistics for each independent signal conditioned on all other signals for the same gene. We used a threshold of $P<1.0\times10^{-5}$ to identify eQTL associations. For each TWAS gene at a psoriasis susceptibility locus, we performed separate colocalization analysis for each 'independent psoriasis signal'–'independent eQTL signal' pair, using the 'coloc' R package (v5.2.2).[29] For example, if a TWAS gene had three independent eQTL signals in blood and is located at a psoriasis susceptibility locus with two independent association signals, we would perform n_{pairs} = 3×2 = 6 colocalization analyses for that gene in blood. For each TWAS gene, we considered a posterior probability for coloc hypothesis 3 (PP3, which supports that association signals are caused by two different SNPs) of >0.5 in all n_{pairs} colocalization tests to constitute evidence of LD contamination (i.e., fully separated psoriasis and eQTL signals). Note that these analyses could only be performed for loci where we had generated COJO conditional association statistics for our psoriasis results, corresponding to loci with at least one genome-wide significant variant having N_{eff} > 93,252 (90% of maximum possible). We also identified TWAS associations with more stringent positive evidence for colocalization between psoriasis susceptibility and eQTL signals. Namely, we report the maximum posterior probability for coloc hypothesis 4 (PP4, which supports that association signals are caused by the same SNP) across all n_{pairs} colocalization tests for each TWAS gene.

Mendelian randomization

For the twelve causal relationships identified using the LCV method, we subsequently performed bidirectional two-sample Mendelian randomization. GWAS summary statistics were obtained for each of the twelve traits.[30-32] In the TwoSampleMR package (v0.5.8)[33], we

used default settings to determine LD-independent genetic instruments at genome-wide significance ($P<5\times10^{-8}$), or at suggestive significance ($P<5\times10^{-8}$) for traits with no genome-wide significant associations. We report Wald ratio causal estimates for instruments comprising a single genetic variant, and inverse variance weighted causal estimates for instruments comprising more than one genetic variant.

Data tabulation/visualisation

Additional basic statistical analyses and tabulation/visualisation of data were performed using R software (version 3.6.3)[34] including packages reshape2 (v.4.4)[35], gridExtra (v2.3)[36], ggrepel (v0.9.1)[37], ggpubr (v0.5.0)[38], ggplot2 (v3.4.0)[39] and data.table (v1.12.8)[40].

Supplementary Note

Definition and number of psoriasis susceptibility loci

We defined a psoriasis susceptibility locus as an LD block (see Supplementary Methods) in which at least one variant exhibited genome-wide significant evidence of association with psoriasis ($P<5\times10^{-8}$) in the full meta-analysis. We considered a psoriasis susceptibility locus (LD block) to have been previously reported in Europeans if it contained the lead variant for one of the 65 association signals reported by previous studies.[1, 41, 42] Note that two of the previously reported association signals did not reach genome-wide significance in the current meta-analysis (as highlighted in main results) and that four of our loci (LD blocks) contained lead variants for two previously reported association signals (Supplementary Data 2). Therefore, 63 of the 65 previously reported association signals map to 59 loci in the current study. We found a further 50 susceptibility loci (LD blocks) that did not overlap with any of the 65 previously reported association signals. We consider these loci to be newly reported in European-ancestry populations. The 59 previously reported and 50 newly reported loci give a total of 109 susceptibility loci in Europeans. One of these loci encompasses the MHC region, which has been shown to include multiple independent association signals[43] and which we did not interrogate further in this study. We found that 27 of the remaining 108 loci contained multiple independent susceptibility signals, including all four loci that mapped to two of the 65 previously reported association signals.

References for Supplementary material

1. Tsoi, L. C. *et al*. Large scale meta-analysis characterizes genetic architecture for common psoriasis associated variants. *Nat. Commun.* **8**, 15382 (2017).

2. Illumina Inc. GenomeStudio Software. https://emea.illumina.com/products/by-type/informatics-products/microarray-software/genomestudio.html.

3. Thermo Fisher Scientific Inc. Affymetrix Power Tools. https://www.thermofisher.com/us/en/home/life-science/microarray-analysis/microarray-analysis-partners-programs/affymetrix-developers-network/affymetrix-power-tools.html.

4. Chang, C. C. *et al*. Second-generation PLINK: rising to the challenge of larger and richer datasets. *Gigascience* **4**, 7–8. eCollection 2015 (2015).

5. Manichaikul, A. *et al*. Robust relationship inference in genome-wide association studies. *Bioinformatics* **26**, 2867–2873 (2010).

6. Jun, G. *et al*. Detecting and estimating contamination of human DNA samples in sequencing and array-based genotype data. *Am. J. Hum. Genet.* **91**, 839–848 (2012).

7. Abraham, G., Qiu, Y. & Inouye, M. FlashPCA2: principal component analysis of Biobank-scale genotype datasets. *Bioinformatics* **33**, 2776–2778 (2017).

8. Loh, P. *et al*. Reference-based phasing using the Haplotype Reference Consortium panel. *Nat. Genet.* **48**, 1443–1448 (2016).

9. Das, S. *et al*. Next-generation genotype imputation service and methods. *Nat. Genet.* **48**, 1284–1287 (2016).

10. Browning, S. R. & Browning, B. L. Rapid and accurate haplotype phasing and missing-data inference for whole-genome association studies by use of localized haplotype clustering. *Am. J. Hum. Genet.* **81**, 1084–1097 (2007).

11. Browning, B. L., Zhou, Y. & Browning, S. R. A One-Penny Imputed Genome from Next-Generation Reference Panels. *Am. J. Hum. Genet.* **103**, 338–348 (2018).

12. Durbin, R. Efficient haplotype matching and storage using the positional Burrows-Wheeler transform (PBWT). *Bioinformatics* **30**, 1266–1272 (2014).

13. Howie, B. N., Donnelly, P. & Marchini, J. A flexible and accurate genotype imputation method for the next generation of genome-wide association studies. *PLoS Genet.* **5**, e1000529 (2009).

14. Marchini, J., Howie, B., Myers, S., McVean, G. & Donnelly, P. A new multipoint method for genome-wide association studies by imputation of genotypes. *Nat. Genet.* **39**, 906–913 (2007).

15. Zhou, W. *et al*. Efficiently controlling for case-control imbalance and sample relatedness in large-scale genetic association studies. *Nat. Genet.* **50**, 1335–1341 (2018).

16. Ellinghaus, E. *et al*. Genome-wide association study identifies a psoriasis susceptibility locus at TRAF3IP2. *Nat. Genet.* **42**, 991–995 (2010).

17. Zou, Y., Carbonetto, P., Wang, G. & Stephens, M. Fine-mapping from summary data with the "Sum of Single Effects" model. *PLoS Genet.* **18**, e1010299 (2022).

18. Wang, G., Sarkar, A., Carbonetto, P. & Stephens, M. A simple new approach to variable selection in regression, with application to genetic fine mapping. *J. R. Stat. Soc. Series B. Stat. Methodol.* **82**, 1273–1300 (2020).

19. Prive, F. Optimal linkage disequilibrium splitting. *Bioinformatics* **38**, 255–256 (2021).

20. Privé, F. 1000 genomes (phase 3) files with SNPs in common with HapMap3 and UK Biobank. https://doi.org/10.6084/m9.figshare.9208979.v3 (2019).

21. 1000 Genomes Project Consortium *et al*. A global reference for human genetic variation. *Nature* **526**, 68–74 (2015).

22. Prive, F., Luu, K., Blum, M. G. B., McGrath, J. J. & Vilhjalmsson, B. J. Efficient toolkit implementing best practices for principal component analysis of population genetic data. *Bioinformatics* **36**, 4449–4457 (2020).

23. Berisa, T. LDetect data. https://bitbucket.org/nygcresearch/ldetect-data/src/master/EUR/ (2015).

24. Berisa, T. & Pickrell, J. K. Approximately independent linkage disequilibrium blocks in human populations. *Bioinformatics* **32**, 283–285 (2016).

25. Karolchik, D. *et al*. The UCSC Table Browser data retrieval tool. *Nucleic Acids Res.* **32**, 493 (2004).

26. Yang, J. *et al*. Conditional and joint multiple-SNP analysis of GWAS summary statistics identifies additional variants influencing complex traits. *Nat. Genet.* **44**, 369–3 (2012).

27. Barbeira, A. N. *et al*. Exploring the phenotypic consequences of tissue specific gene expression variation inferred from GWAS summary statistics. *Nat. Commun.* **9**, 1825–1 (2018).

28. GTEx Consortium *et al*. Genetic effects on gene expression across human tissues. *Nature* **550**, 204–213 (2017).

29. Giambartolomei, C. *et al*. Bayesian test for colocalisation between pairs of genetic association studies using summary statistics. *PLoS Genet.* **10**, e1004383 (2014).

30. Neale Lab. UK Biobank GWAS round 2. http://www.nealelab.is/uk-biobank/.

31. Willer, C. J. *et al*. Discovery and refinement of loci associated with lipid levels. *Nat. Genet.* **45**, 1274–1283 (2013).

32. Haworth, S. GWAS summary statistics for dental caries and periodontitis. https://doi.org/10.5523/bris.2j2rqgzedxlq02oqbb4vmycnc2 (2019).

33. Hemani, G. *et al*. The MR-Base platform supports systematic causal inference across the human phenome. *Elife* **7**, 10.7554/eLife.34408 (2018).

34. R Core Team. R: A Language and Environment for Statistical Computing. (R Foundation for Statistical Computing, 2024). https://www.R-project.org/.

35. Wickham, H. Reshaping Data with the reshape Package. *J. Stat. Soft.* **21**, 1 (2007).

36. Auguie, B. gridExtra: Miscellaneous Functions for "Grid" Graphics (2021). https://CRAN.R-project.org/package=gridExtra.

37. Slowikowski, K. ggrepel: Automatically Position Non-Overlapping Text Labels with 'ggplot2' (2021). https://CRAN.R-project.org/package=ggrepel.

38. Kassambara, A. ggpubr: 'ggplot2' Based Publication Ready Plots (2022). https://CRAN.R-project.org/package=ggpubr.

39. Wickham, H. in *ggplot2: Elegant Graphics for Data Analysis* (Springer-Verlag New York, 2016).

40. Dowle, M. & Srinivasan, A. data.table: Extension of `data.frame` (2019). https://CRAN.R-project.org/package=data.table.

41. Dand, N. *et al*. Exome-wide association study reveals novel psoriasis susceptibility locus at TNFSF15 and rare protective alleles in genes contributing to type I IFN signalling. *Hum. Mol. Genet.* **26**, 4301–4313 (2017).

42. Patrick, M. T. *et al*. Genetic signature to provide robust risk assessment of psoriatic arthritis development in psoriasis patients. *Nat. Commun.* **9**, 4178–6 (2018).

43. Stuart, P. E. *et al*. Transethnic analysis of psoriasis susceptibility in South Asians and Europeans enhances fine-mapping in the MHC and genomewide. *HGG Adv.* **3**, 10.1016/j.xhgg.2021.100069. Epub 2021 Nov 6 (2022).

Supplementary Figure 1 – Association results across the extended MHC region

x-axis, position on chromosome 6; y-axis, -log10(P-value) of association (two-sided *Z*-test, unadjusted for multiple tests); blue points, variants within the extended MHC region as defined by Horton et al. (Nat Rev Genet, 2004); orange points, variants in an adjacent region of genome-wide significant association outside the Horton-delimited MHC. These signals all occur within the same LD block and as such are counted as a single susceptibility locus, although the MHC is known to harbour multiple independent psoriasis susceptibility signals.

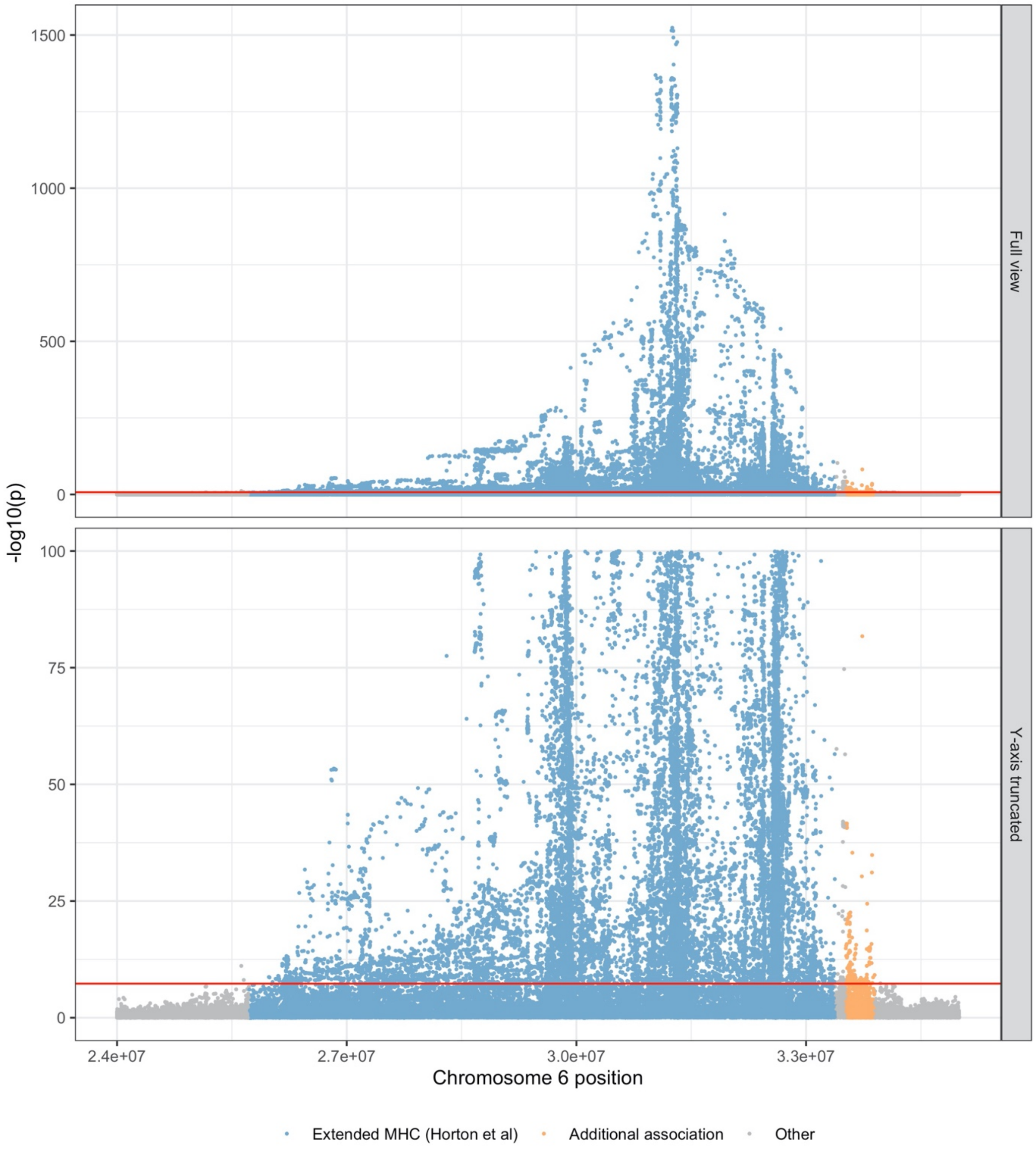

Supplementary Figure 2 – Effect size consistency vs 2017 meta-analysis

Points represent 63 lead variants in 2017 psoriasis susceptibility meta-analysis (Tsoi et al, Nat Commun 2017). Grey bars, 95% confidence intervals; x-axis, reference allele effect estimated in 2017 meta-analysis; blue line, *x*=*y*. *Left panel*: y-axis, reference allele effect estimated in the current study. *Right panel*: y-axis, reference allele effect estimated by meta-analysing the 12 GWAS datasets in the current study that were not previously included in the 2017 meta-analysis (excluded datasets: CASP, ExomeChip, Genizon, Kiel, PsA, WTCCC2).

The point overlapping the x-axis in the left panel represents rs10789285 (chr1:69788482), identified at genome-wide significance in an earlier study (Tsoi et al, Nat Commun 2015) and nominally significant in the 2017 study (P_{2017} = 1.6×10^{-4}). It is not significant in the current study (P_{meta} = 0.510), but falls within an LD block (chr1:65151018-76975228) with three independent susceptibility signals (Supplementary Data 1), each strongly associated with psoriasis risk (joint model P_{meta} = 1.55×10^{-47}, 3.91×10^{-27} and 3.61×10^{-17} respectively).

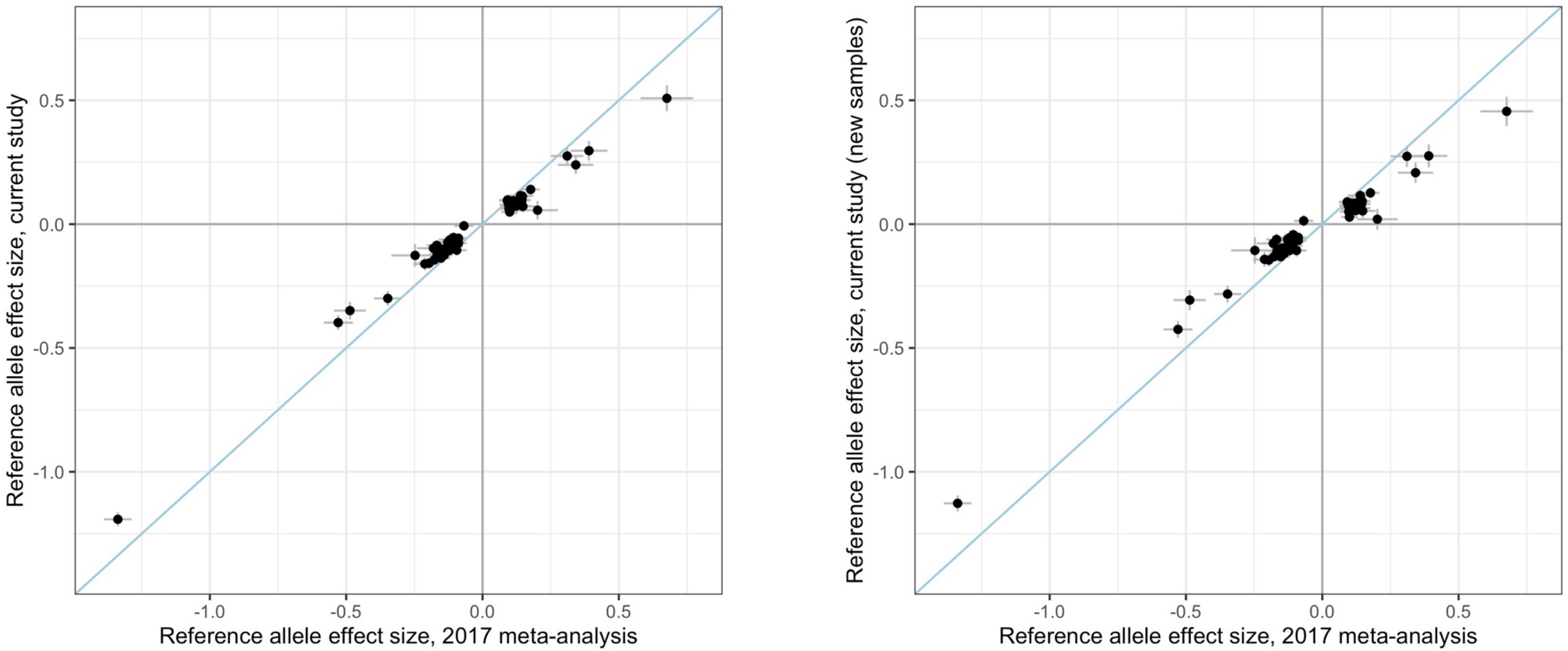

Supplementary Figure 3 – Effect size heterogeneity at lead variants

Points represent lead variants at 109 susceptibility loci. Point colour indicates outcome of heterogeneity test (Cochran's *Q*), denoting nominal (P_{het}<0.05) or Bonferroni-corrected (P_{het}<4.6×10^{-4}) significance. I^2, estimated fraction of variance due to heterogeneity.

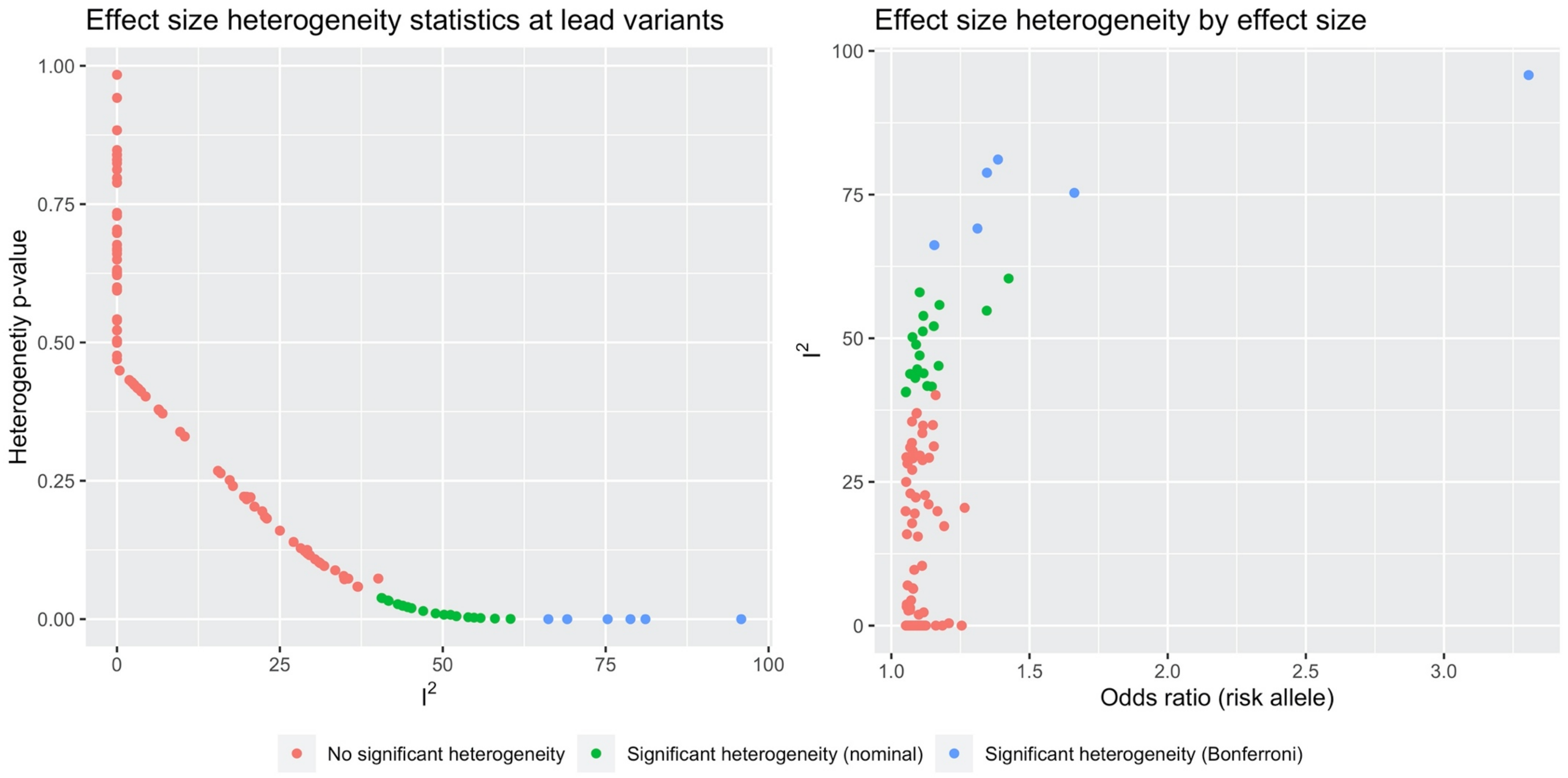

Supplementary Figure 4 – Effect sizes at lead variants with significant heterogeneity

Forest plots of effect sizes (x-axis) for each GWAS study (y-axis) for the six lead variants at loci with Bonferroni-significant effect size heterogeneity. Studies ordered by effect size. Error bars: 95% confidence interval; point color: study type.

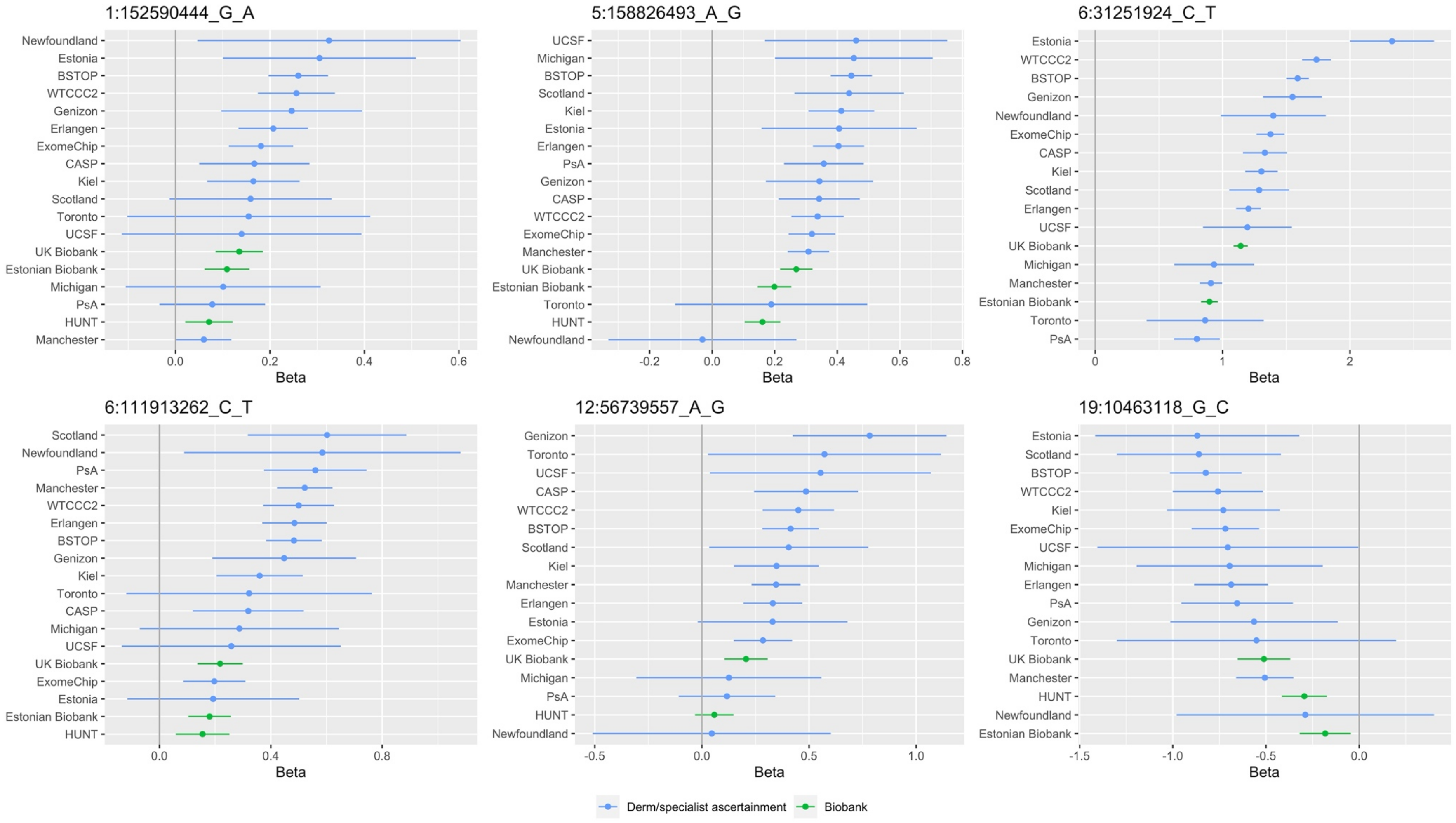

Supplementary Figure 5 – Comparison of 95% Bayesian credible set length to previous GWAS meta-analysis

Comparison of 95% Bayesian credible set (BCS) lengths to previous GWAS meta-analysis. Each point represents a different association signal established in the previous meta-analysis (Tsoi et al., 2017). Point colour indicates direction of change, blue dashed line indicates equality.

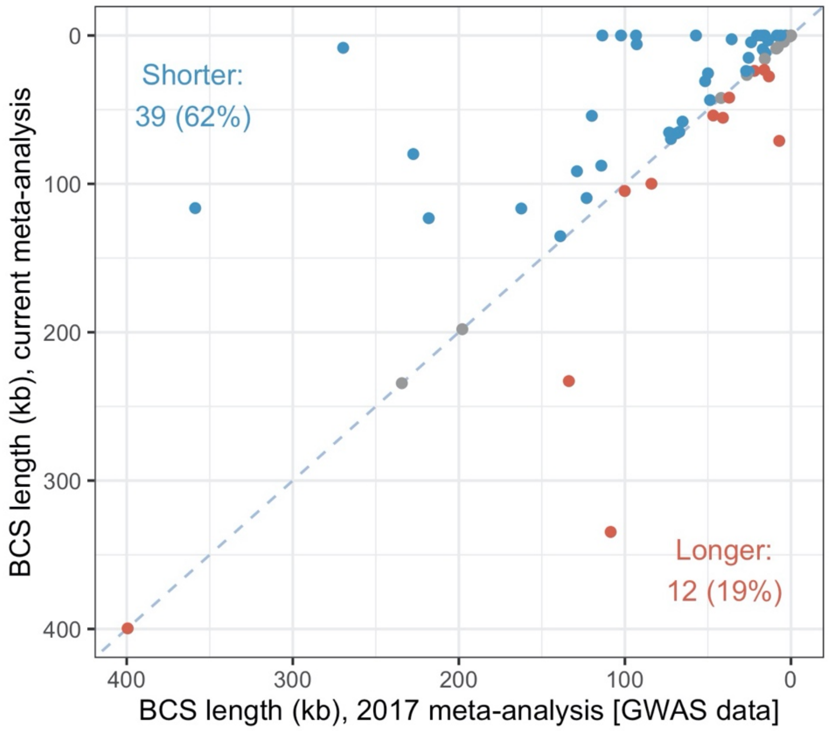

Supplementary Figure 6 – Frequency-effect size characteristics of known and novel loci

Points represent non-MHC susceptibility loci, highlighted by whether a protein-altering variant is included in the 95% Bayesian credible set (diamond if CADD score > 15, circle otherwise). Point color: known vs novel susceptibility locus; x-axis: lead variant minor allele frequency (estimated from 1000 Genomes EUR data); y-axis: lead variant risk allele effect size.

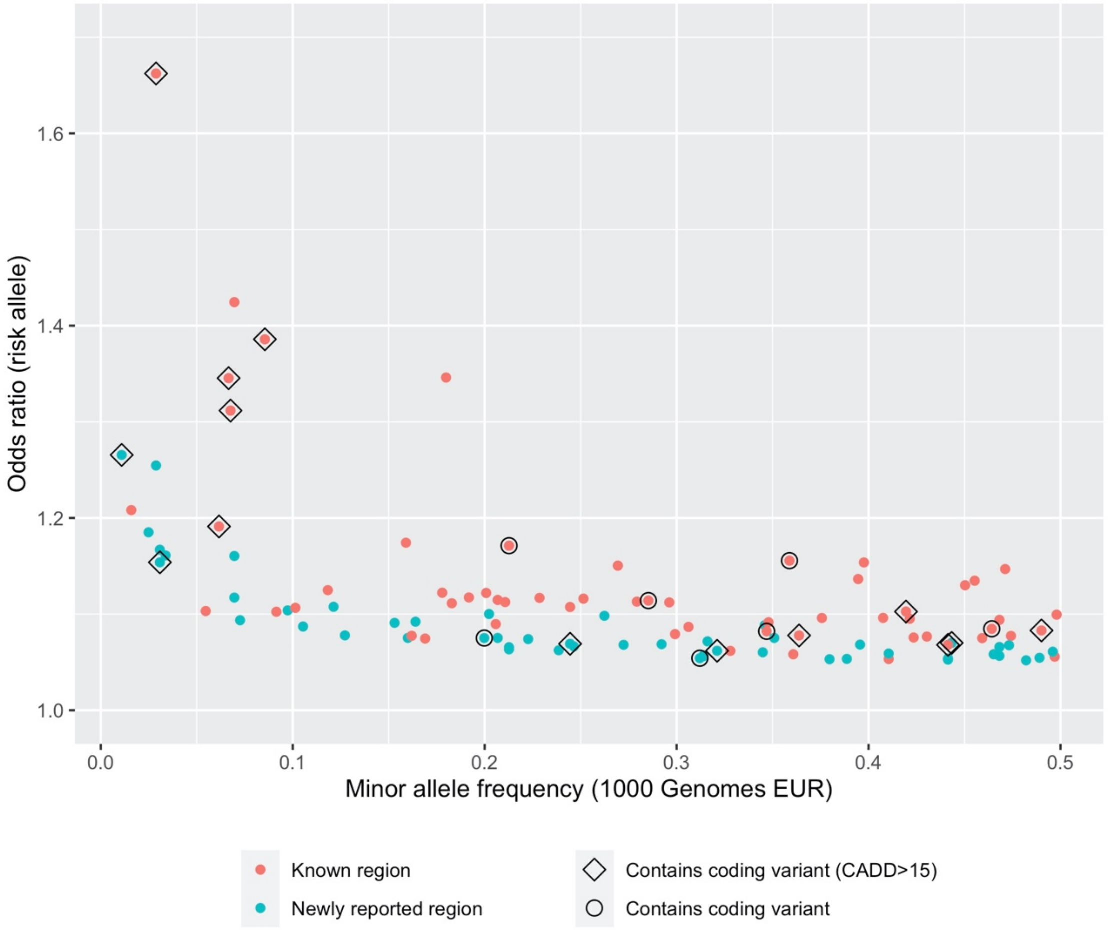

Supplementary Figure 7 – Credible set enrichment for predicted regulatory variants

Variant regulatory probabilities estimated by TURF (y-axis), summarised by size of Bayesian credible set (x-axis). Upper boxplot: TURF generic probabilities; lower boxplot: maximum of skin-specific and blood-specific regulatory probability is used for each variant. Boxes represent interquartile range (IQR) with horizontal bar at median. Whiskers indicate full range of data except for outlying variants (>1.5× IQR from box). Individual data points are shown for all variants (total *n*=5,102 credible set variants corresponding to *n*=143 independent psoriasis susceptibility signals). BCS, Bayesian credible set.

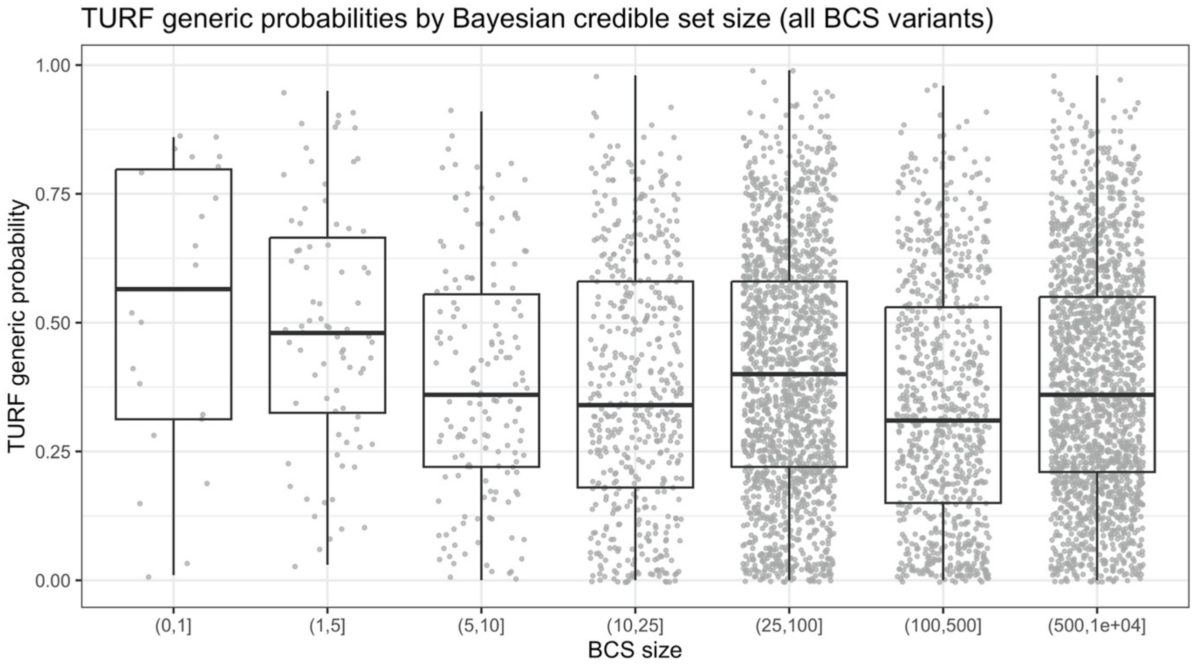

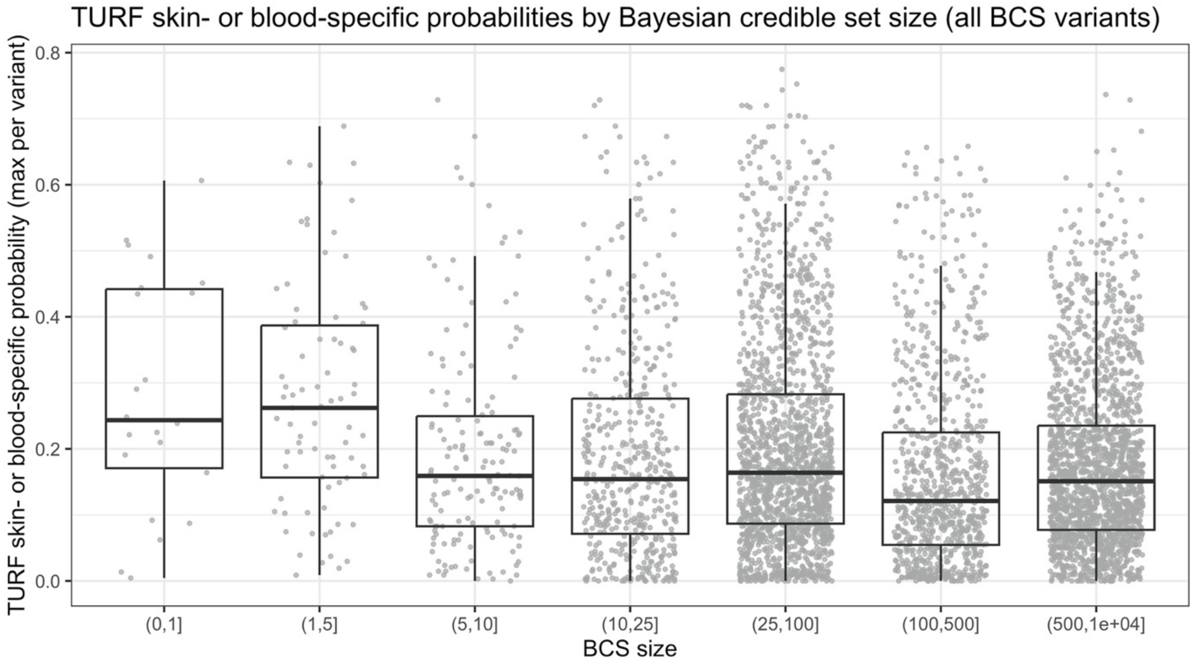

Supplementary Figure 8 – High-confidence regulatory variants identified by TURF analysis

For the 14 candidate regulatory variants, plots show generic (black points) and tissue-specific (grey) regulatory probabilities (y-axis) estimated by TURF for all 51 tissues (x-axis). Blood and skin are highlighted in orange and blue, respectively (inverted triangles for ease of identification).

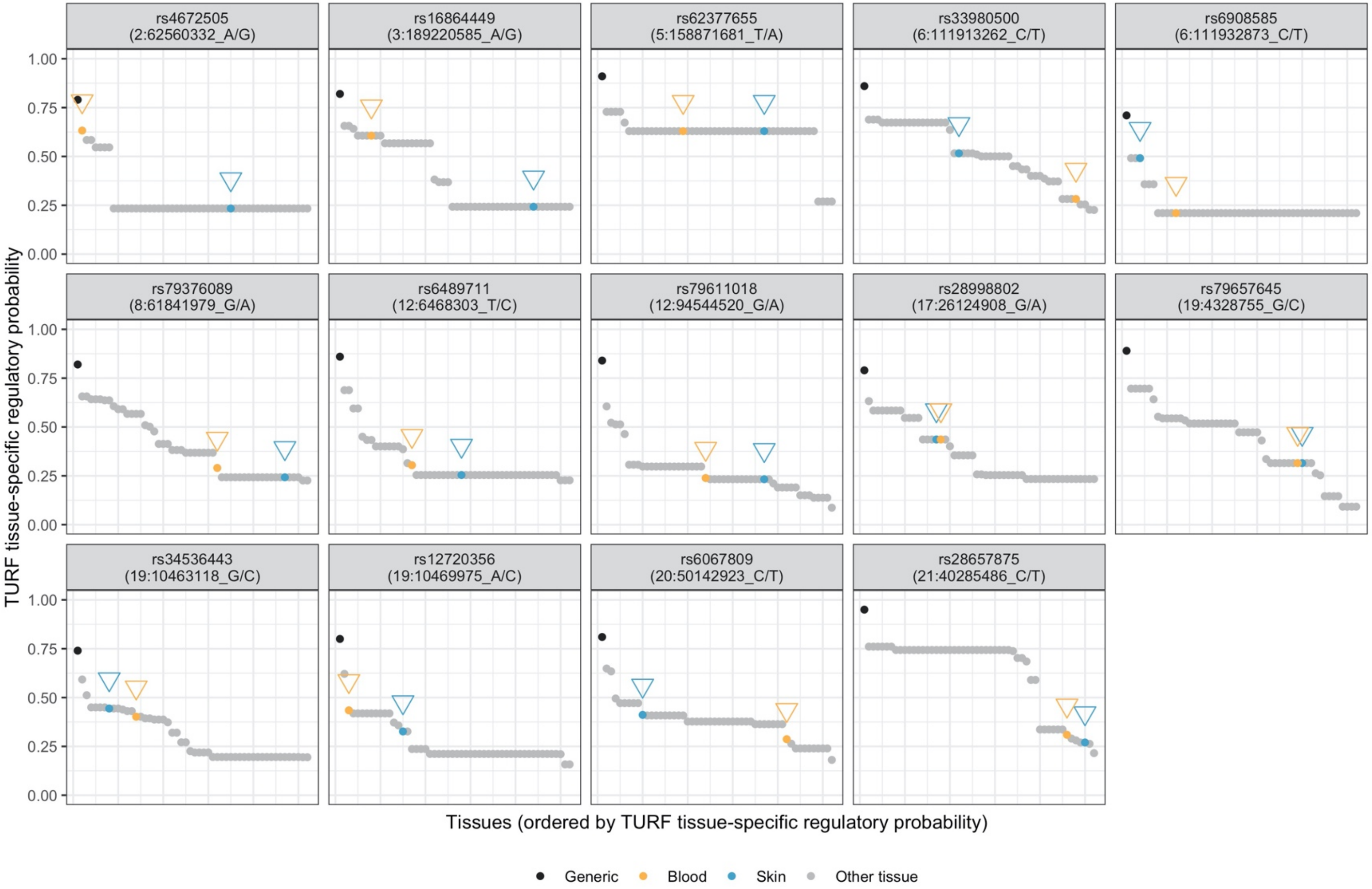

Supplementary Figure 9 – Manhattan plots for transcriptome-wide association study

Each point represents a gene with predicted expression; point colours for significant TWAS genes indicate whether gene is subsequently filtered out due to poor model prediction p-value or coloc “PP3” evidence that psoriasis and eQTL signals are independent (see Methods), otherwise colours alternate by chromosome with MHC region genes on chromosome 6 greyed out; x-axis: position in genome; y-axis: -$\log_{10}$(TWAS association p-value) (two-sided *Z*-test, unadjusted for multiple tests); horizontal orange line: transcriptome-wide significance threshold (2.18×10^{-6}); panels represent predicted expression results in different tissues (sun-unexposed skin, sun-exposed skin, whole blood).

A – All results

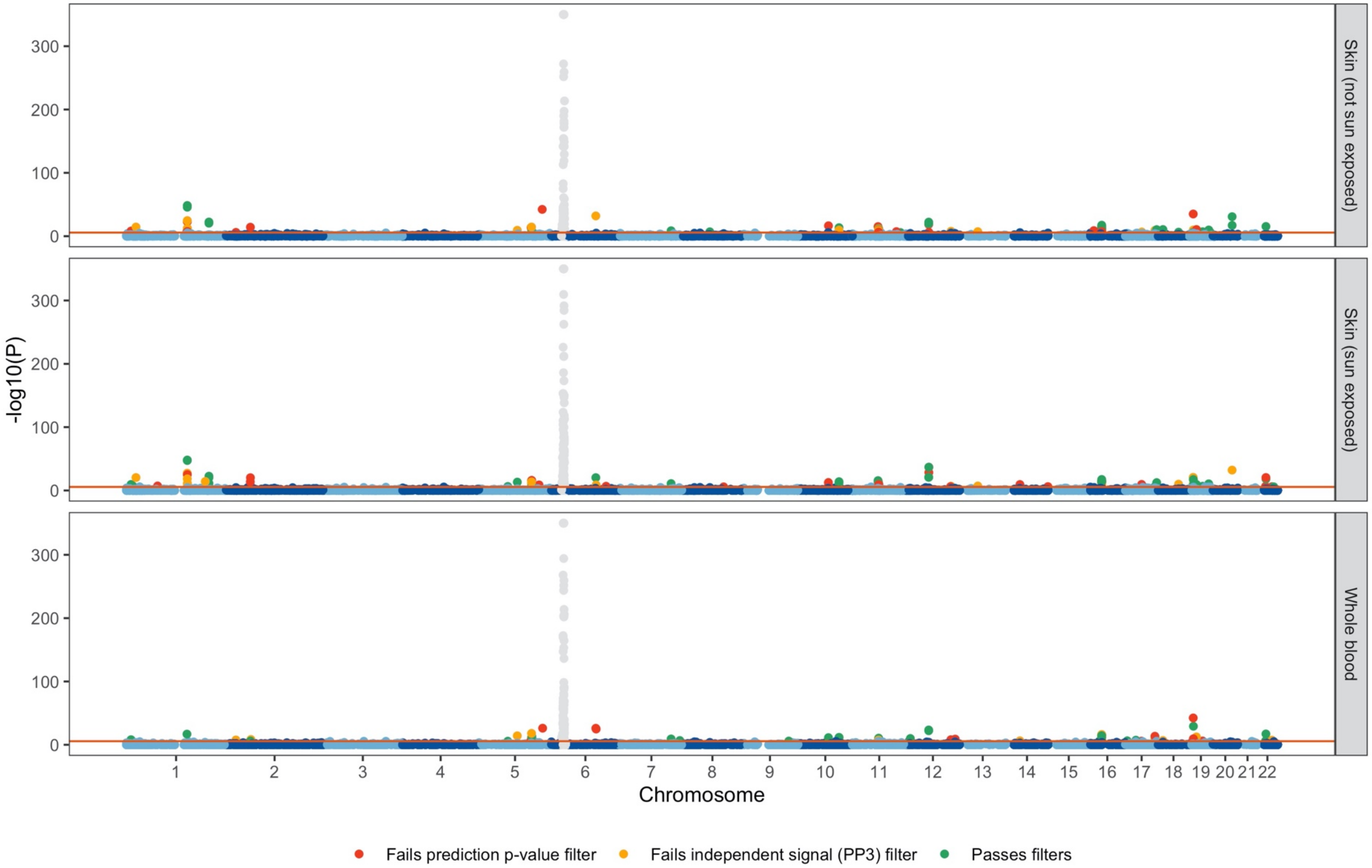

B – Truncated y-axis for clearer resolution at non-MHC signals

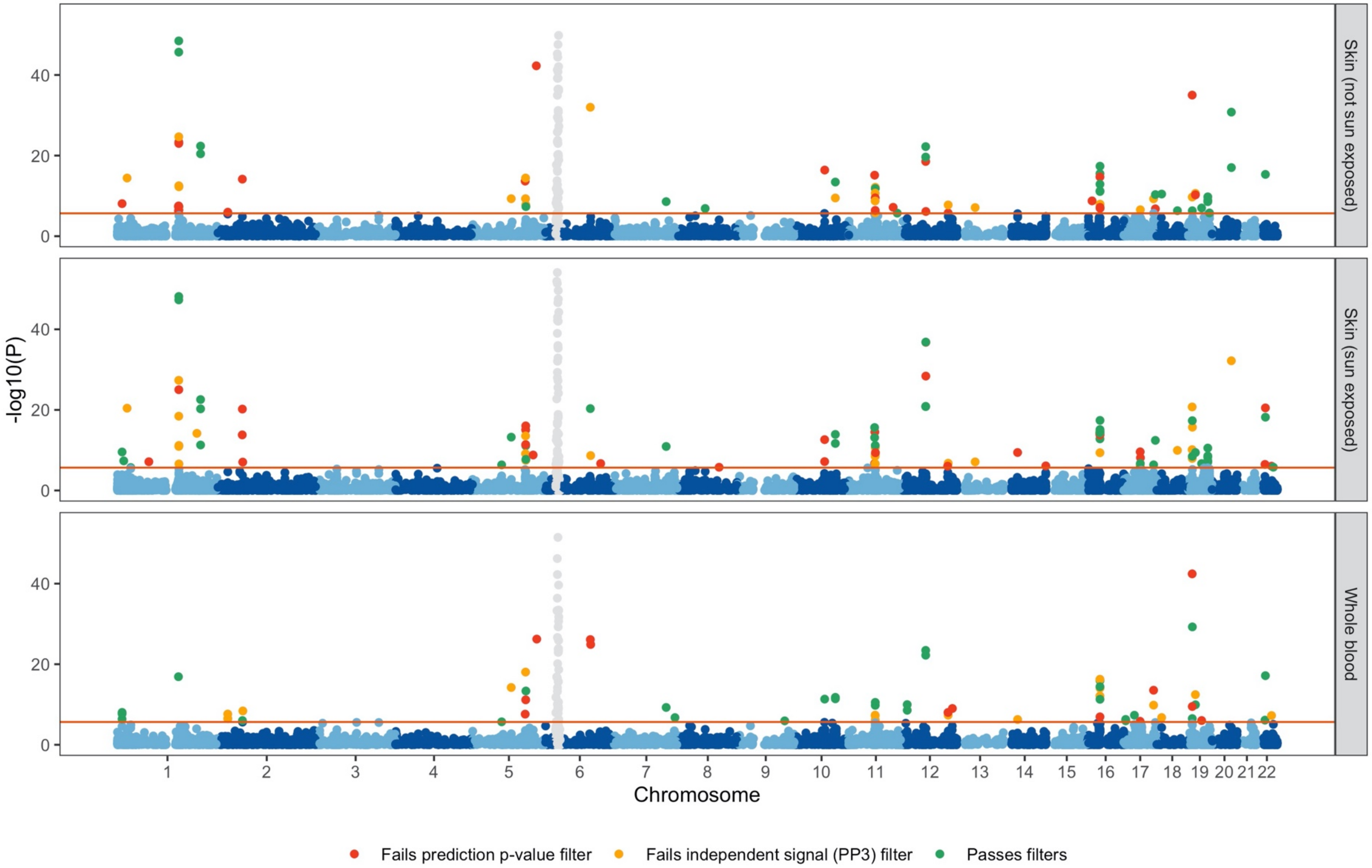

Supplementary Figure 10 – Physical location of significant TWAS genes

Genes allocated to each of 108 non-MHC psoriasis susceptibility regions (see Methods) are plotted by position (of their transcription start site) relative to the lead meta-analysis variant; x-axis: position relative to lead variant; y-axis: one row per genomic region; vertical tick marks indicate lower and upper limits of each LD block; orange and green points indicate transcriptome-wide significant genes that fail and pass subsequent filtering steps, respectively (see Methods); biotype “other” refers to a handful of TWAS genes that are transcribed or processed pseudogenes or encode lincRNAs.

A – LD blocks 1-36

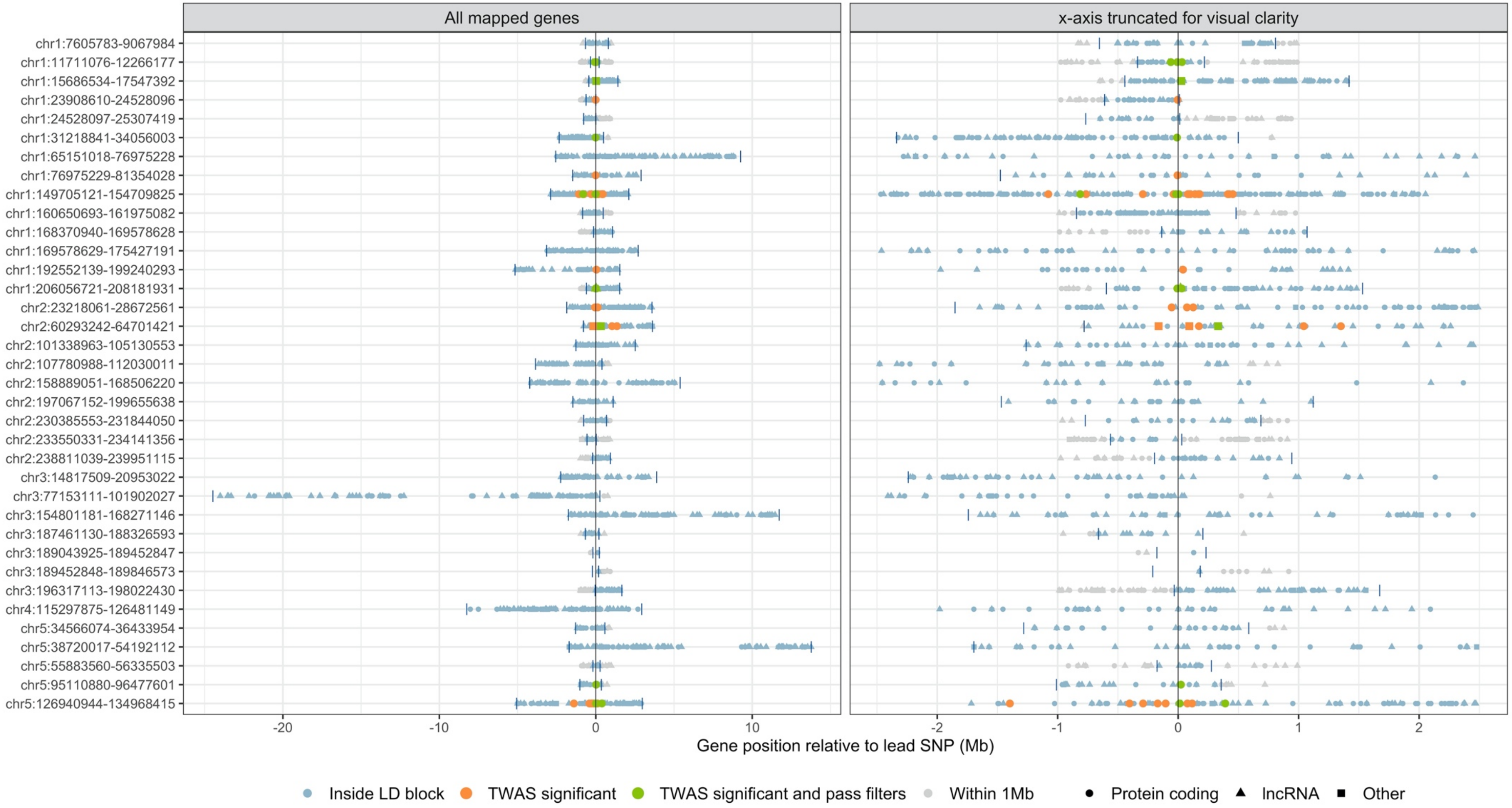

B – LD blocks 37-73 (excludes MHC block 41)

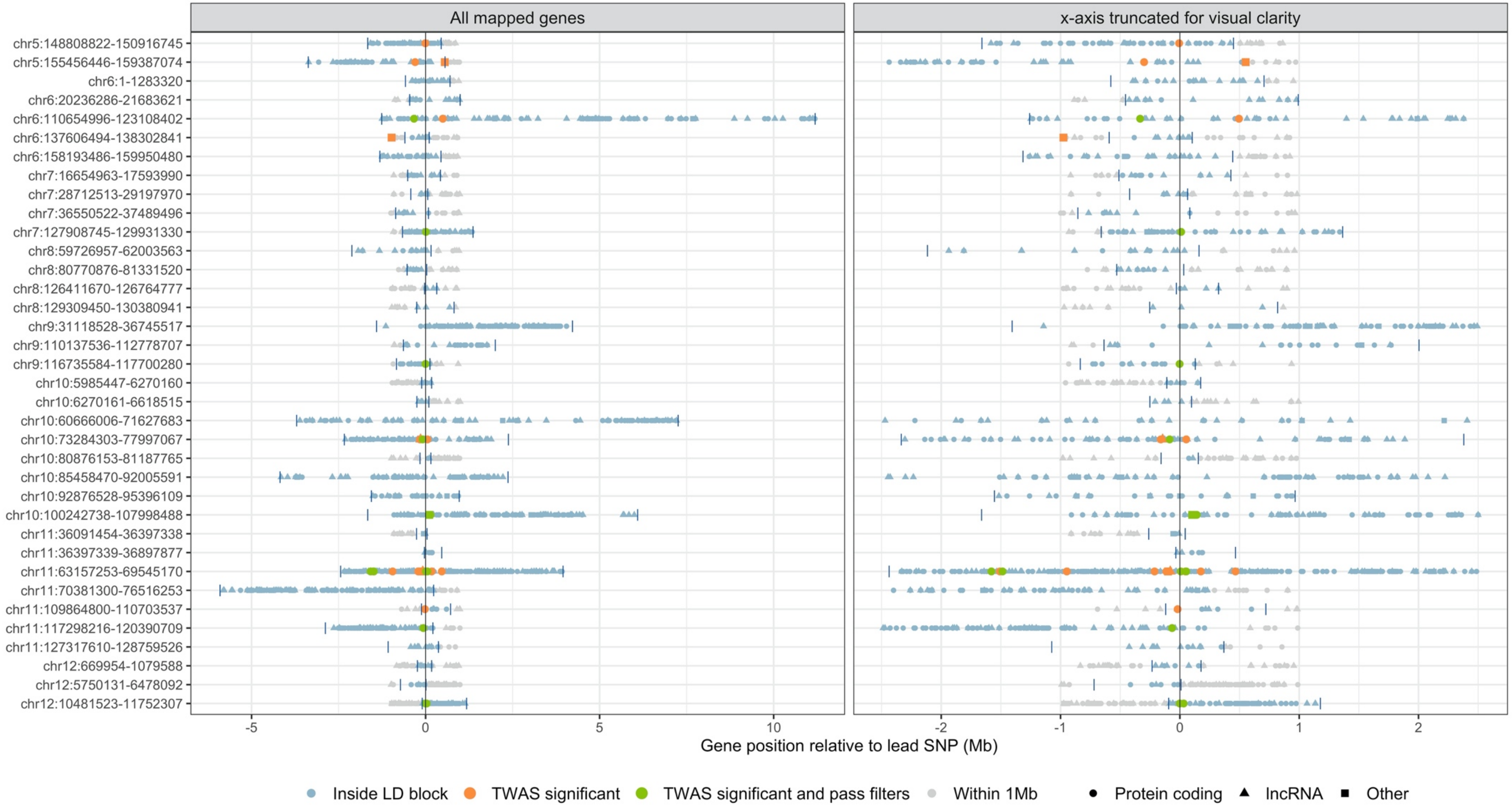

C – LD blocks 74-109

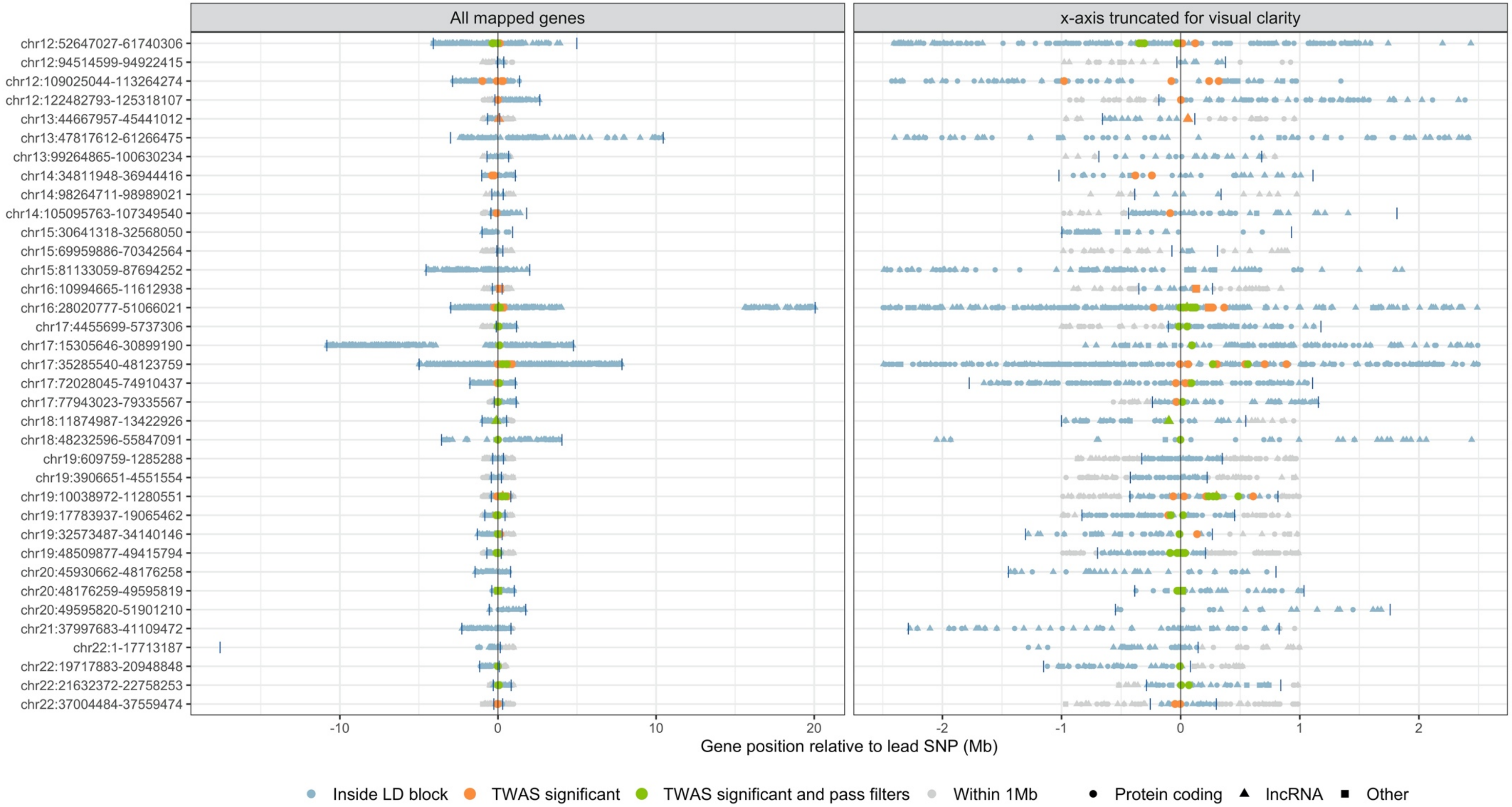

Supplementary Figure 11 – Comparison of gene set enrichment results from DEPICT

Each point represents a gene set (n=14,462); x-axis, rank among all gene sets (A, left-hand plot) or $-\log_{10}$(enrichment p-value) (B, right-hand plot) based on DEPICT analysis using previously reported psoriasis susceptibility regions only; y-axis, rank (left-hand plot) or $-\log_{10}$(enrichment p-value) (right-hand plot) based on DEPICT analysis using all psoriasis susceptibility regions; point colour indicates enrichment status based on false-discovery rate <5%. P-value is empirically derived bias-adjusted Z-score as described in Pers et al, *Nat Commun* 2015.

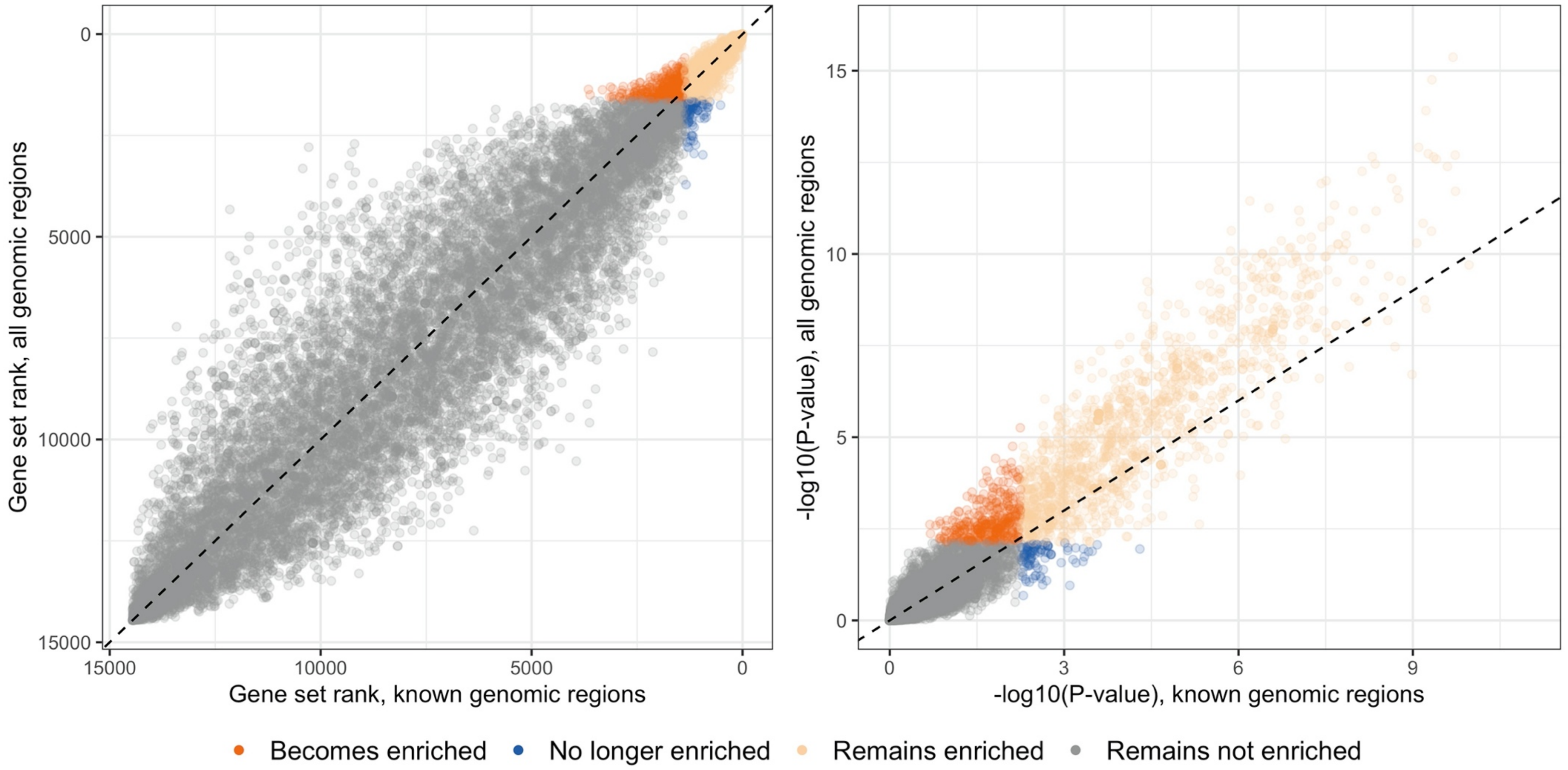

Supplementary Figure 12 – Enrichment for TWAS genes among cytokine-regulated genes in keratinocytes

Points indicate cytokine used to stimulate keratinocytes prior to RNA-seq; y-axis: enrichment (observed:expected ratio) for TWAS target genes among the set of genes significantly induced after cytokine stimulation; x-axis: statistical significance of enrichment (measured as $-\log_{10}$(FDR), derived from one-sided hypergeometric tests). Coincident points are overplotted, with multiple (bracketed) labels.

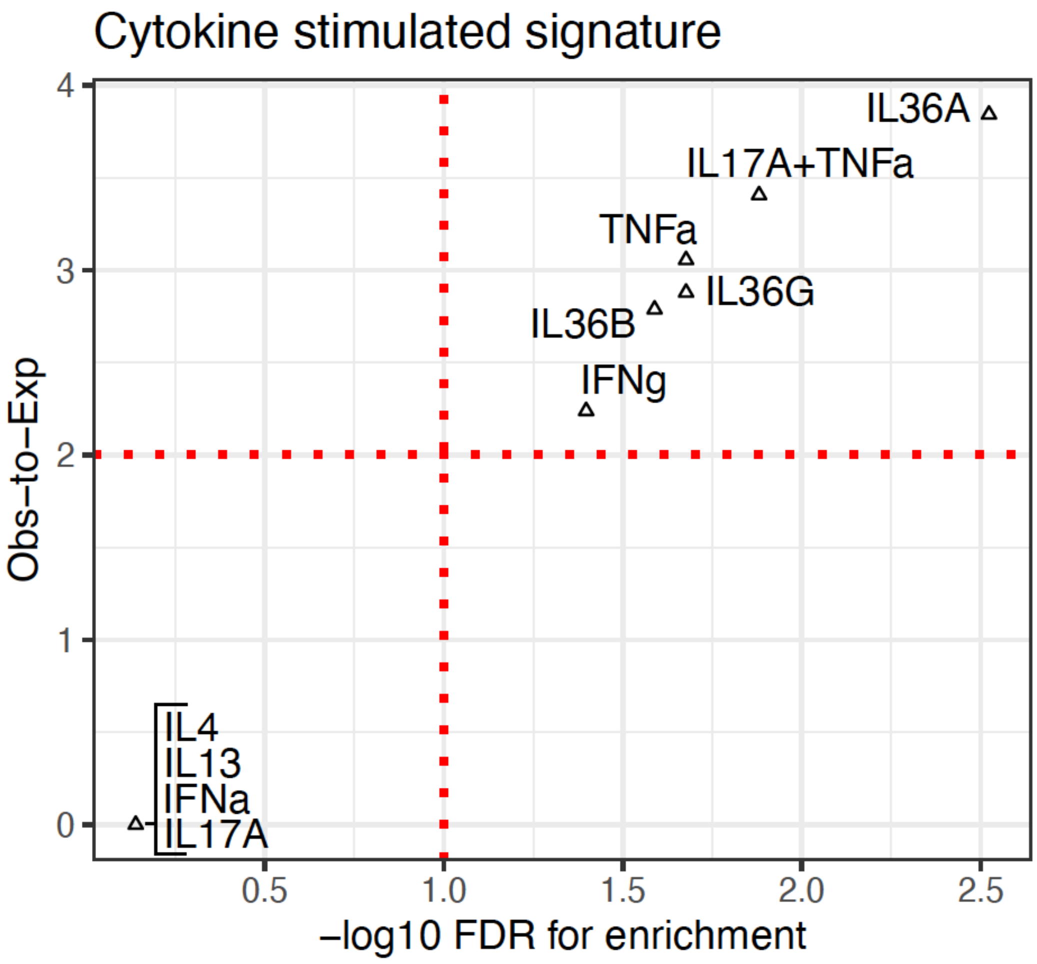

Supplementary Figure 13 – Genetic correlations with disease and health traits

Largest estimated genetic correlations with psoriasis. Up to ten traits with statistically significant genetic correlation ($P<8.4\times10^{-5}$) are displayed; x-axis, genetic correlation (r_g); error bars: 95% confidence interval. No negatively correlated disease and health traits reached statistical significance.

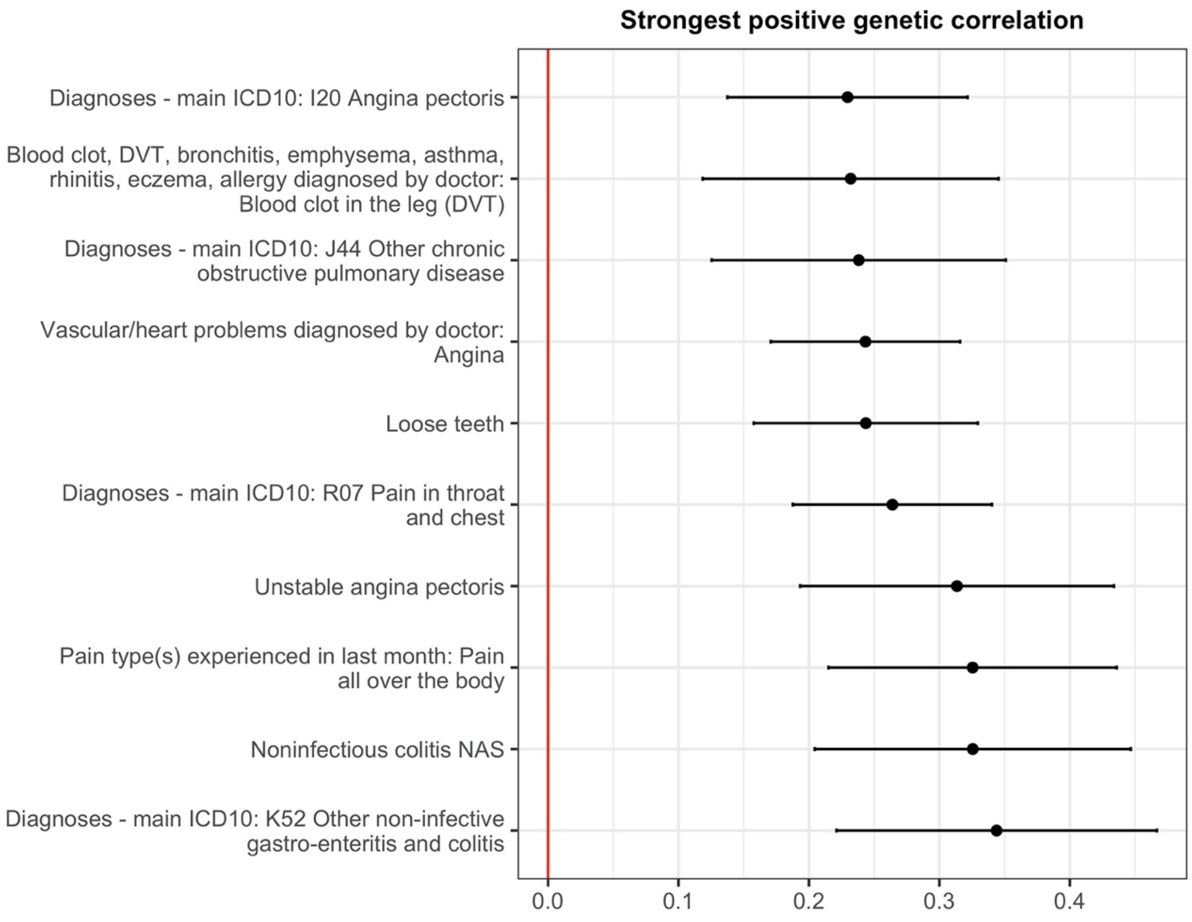

Supplementary Figure 14 – Genetic correlations with physical, cognitive and biochemical measures

Largest estimated negative and positive genetic correlations with psoriasis. Up to ten traits with statistically significant genetic correlation ($P<8.4\times10^{-5}$) are displayed; x-axis, genetic correlation (r_g); error bars: 95% confidence interval.

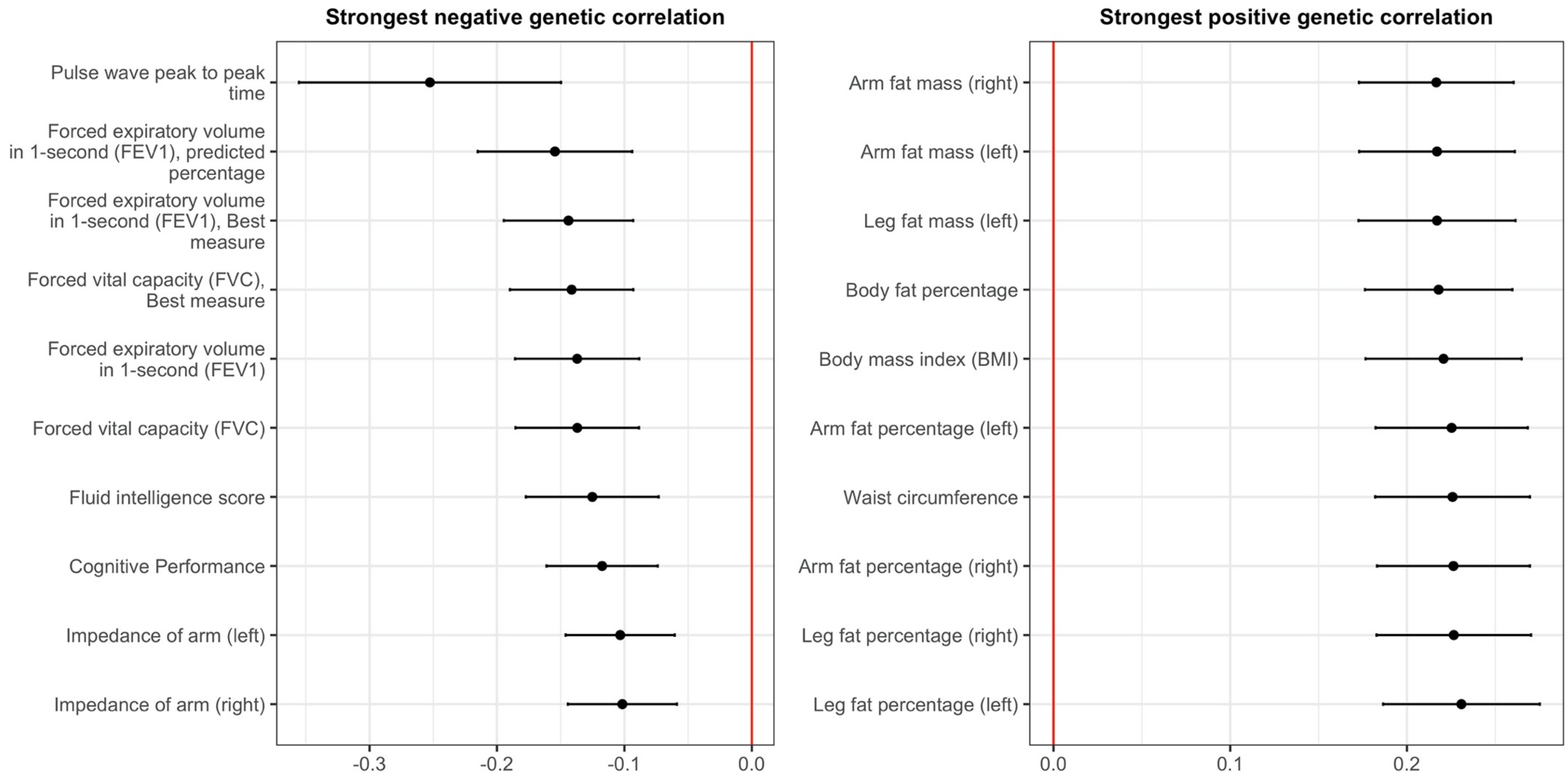

Supplementary Figure 15 – Genetic correlations with lifestyle and quality of life factors

Largest estimated negative and positive genetic correlations with psoriasis. Up to ten traits with statistically significant genetic correlation (P<8.4×10^{-5}) are displayed; x-axis, genetic correlation (r_g); error bars: 95% confidence interval.

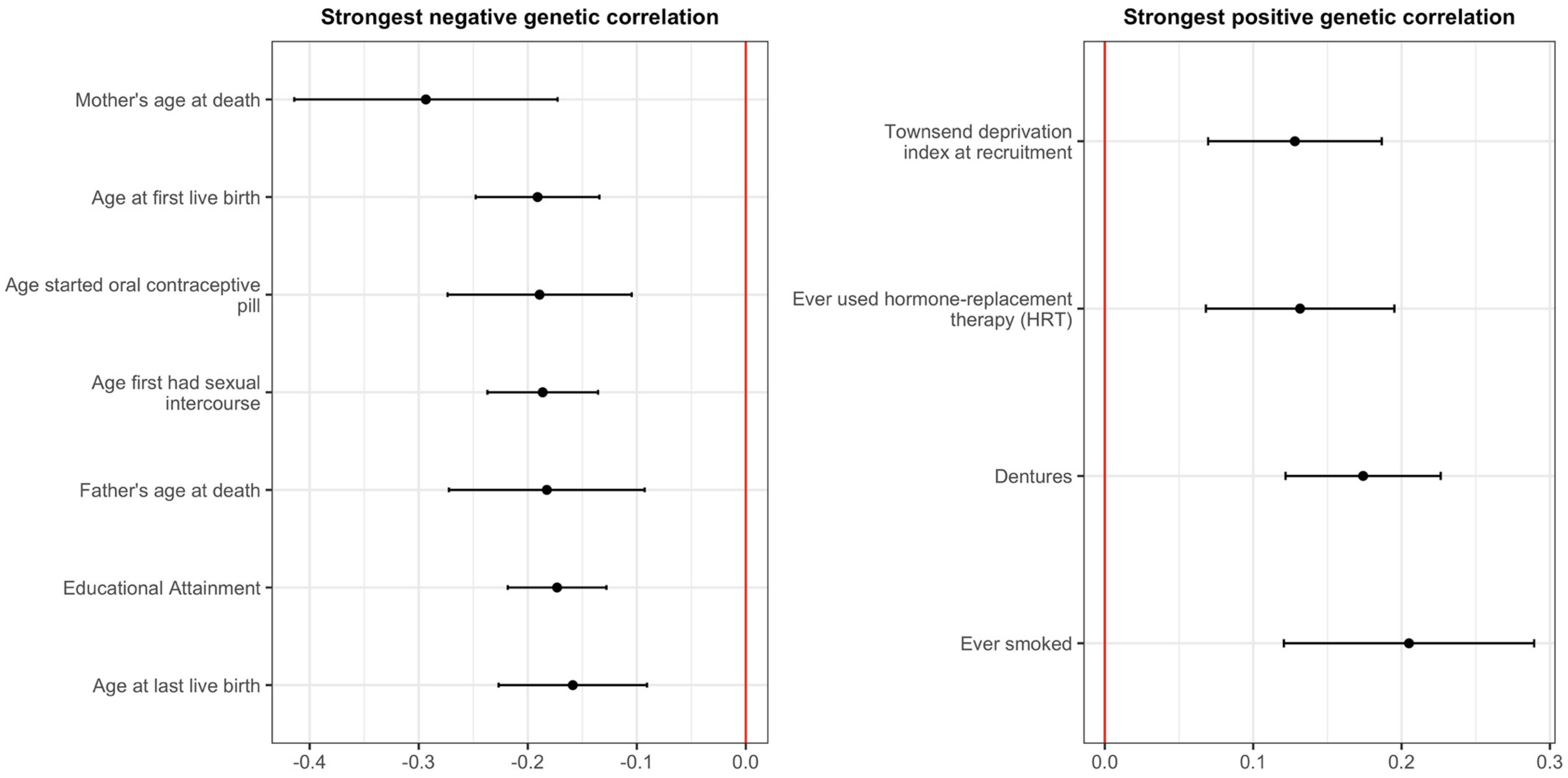

Supplementary Figure 16 – Latent causal variable analysis

Estimated causal architecture between psoriasis and other traits. Each point represents a different trait tested; x-axis, genetic causality proportion (GCP), points to the left of the grey dashed line have a causal influence on psoriasis, points to the right are causally influenced by psoriasis; y-axis: statistical significance (z score) for GCP; point colour, genetic correlation (r_g); point size, magnitude of r_g; traits with a significant GCP at FDR<0.05 lie above the dashed red line and are highlighted with a grey border. Key to numbering on next page.

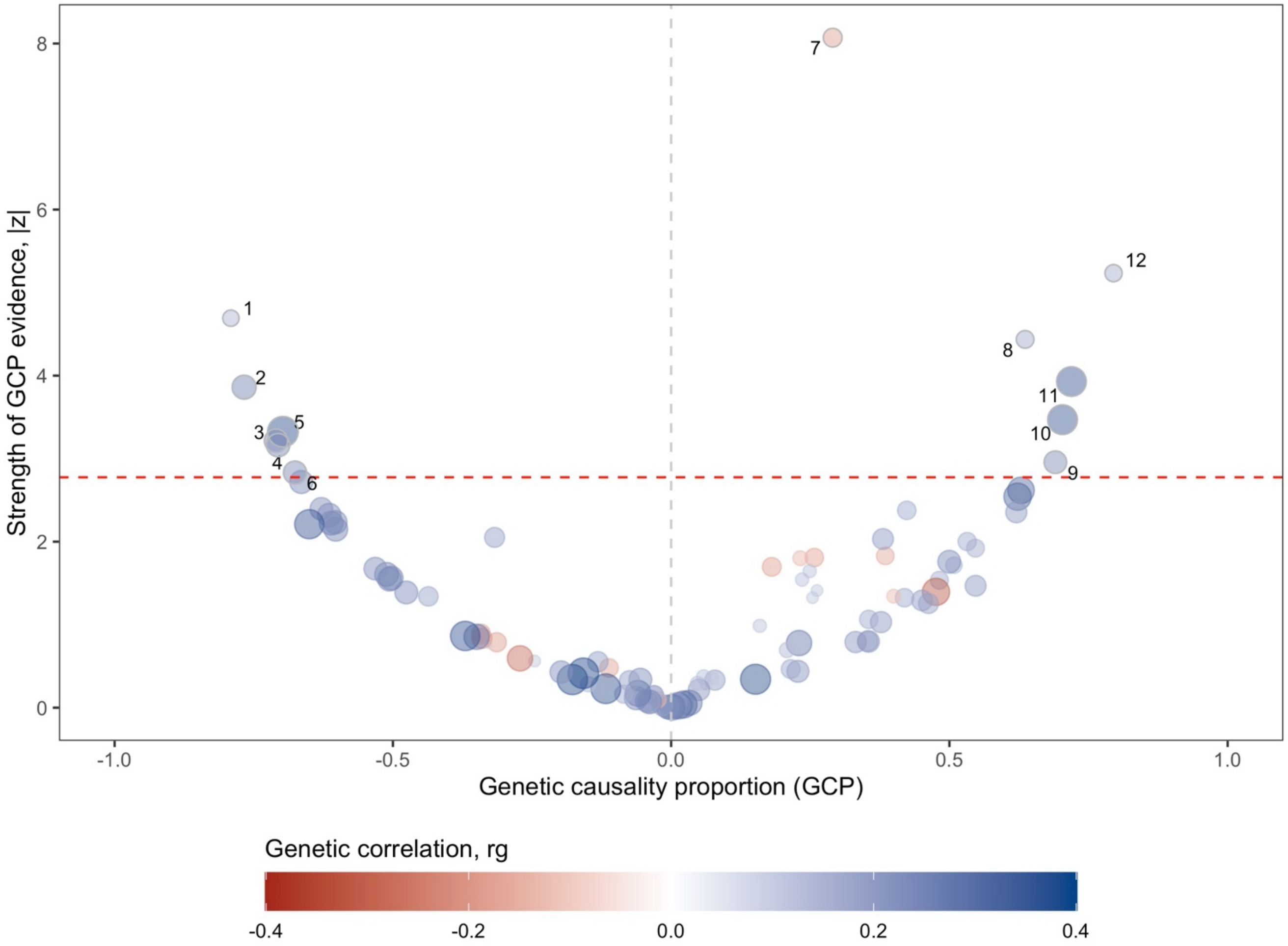

Key for Supplementary Figure 16

Negative GCP (implies trait has causal role in psoriasis)

1. Triglycerides
2. Waist circumference
3. Leg fat mass (right)
4. Leg fat mass (left)
5. Stroke (self-reported)
6. Body mass index (BMI)

Positive GCP (implies psoriasis has causal role in trait)

7. Age started oral contraceptive pill
8. Periodontitis + loose teeth
9. Diabetes (self-reported)
10. Fracture at wrist and hand level (hospital in-patient diagnosis)
11. Pain all over the body experienced in last month
12. Back pain experienced in last month

Supplementary Figure 17 – Evidence for association at loci previously reported in non-European populations

Points represent most significant previously reported associations at loci outside of meta-analysis loci (LD blocks); x-axis, negative $\log_{10}$ p-value of association reported in the earlier study; point colour, ancestry in which association reported by the earlier study; y-axis, negative $\log_{10}$ p-value for the most strongly associated variant within 500 kb in the current study (two-sided *Z*-test, unadjusted for multiple tests); horizontal and vertical red lines, genome-wide significance threshold (5.0×10^{-8}).

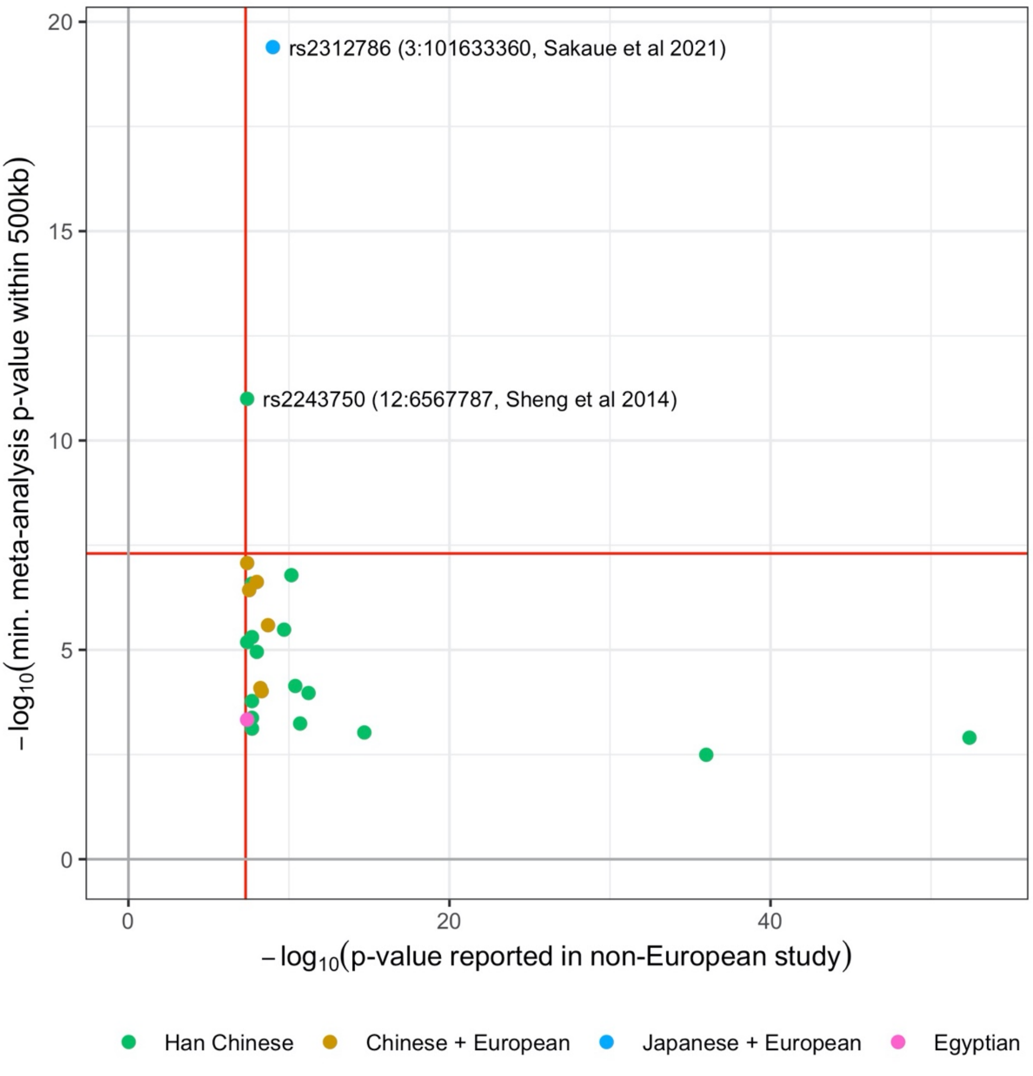

Supplement: Supplementary file 1 — Supplementary Information [file 41467_2025_56719_MOESM1_ESM.pdf]
