# Supplementary material for: GWAS meta-analysis of psoriasis identifies new susceptibility alleles impacting disease mechanisms and therapeutic targets
Source: Nat Commun. 2025 Feb 28;16:2051. doi: 10.1038/s41467-025-56719-8 (PMC11871359; doi:10.1038/s41467-025-56719-8)
Supplement: Supplementary file 2 — Description of Additional Supplementary Files [file 41467_2025_56719_MOESM2_ESM.pdf]

Description of Additional Supplementary Files

Note that all Supplementary Data tables listed below are provided together in the uploaded file 'Psoriasis_meta_resub2_vFINAL_supplementary_data.xlsx'

File Name: Supplementary Data 1
Description: Summary of meta-analysis association signals. Chr, chromosome; LD, linkage disequilibrium; EA, effect allele; NEA non-effect allele; Het., heterogeneity; PP, posterior probability. Psoriasis-associated regions not previously reported in European populations are shaded yellow.

File Name: Supplementary Data 2
Description: Previously reported European psoriasis susceptibility loci not found genome-wide significantly associated. Chr, chromosome; EA, effect allele; NEA non-effect allele; OR, odds ratio; P, meta-analysis p-value (two-sided Z-test, unadjusted for multiple tests).

File Name: Supplementary Data 3
Description: Summary of loci newly reported in Europeans. Chr, chromosome; EA, effect allele; NEA non-effect allele; PP, posterior probability. Newly reported loci with multiple independent association signals are shaded grey.

File Name: Supplementary Data 4
Description: Effect sizes reported in other populations at loci newly reported in Europeans. Chr, chromosome; EA, effect allele; NEA non-effect allele; OR, odds ratio; CI, confidence interval.

File Name: Supplementary Data 5
Description: Variance explained by independent psoriasis susceptibility signals. All inferred independent signals used with estimated joint effect sizes in all regions except for MHC, which used single lead signal with marginal effect (see Methods).

File Name: Supplementary Data 6
Description: Summary of statistical fine-mapping. N_{eff}, cumulative effective sample size; BCS, Bayesian 95% credible set; PP_{max}, maximum posterior probability for causality among variants in BCS.

File Name: Supplementary Data 7
Description: Comparison of 95% Bayesian credible sets to previous GWAS meta-analysis. BCS, Bayesian 95% credible set; PP_{max}, maximum posterior probability for causality among variants in BCS.

File Name: Supplementary Data 8
Description: Protein-altering variants in psoriasis susceptibility regions. $PP_{prot\text{-}alt}$, posterior probability that protein-altering variant is causal for the associated susceptibility signal based on statistical fine-mapping.

File Name: Supplementary Data 9
Description: Enrichment for likely regulatory variants by credible set size. Variant regulatory probabilities estimated by TURF, summarised by size of Bayesian credible set. BCS, Bayesian credible set.

File Name: Supplementary Data 10
Description: Candidate variants with highly probable regulatory function. Variants identified by TURF with high generic regulatory probability (>0.7), high share of regulatory probability across their Bayesian credible set (>50%) and low probability that other variants in the BCS are regulatory (all <0.5). BCS, Bayesian credible set; n/a, not applicable

File Name: Supplementary Data 11
Description: Mediated expression score analysis. Results presented as: Estimate (Standard error). h^2, estimated SNP heritability; h^2_{med}, estimated heritability mediated by cis genetic component of gene expression; GTEx, Genotype-Tissue Expression project.

File Name: Supplementary Data 12
Description: Transcriptome-wide psoriasis-associated genes. Excludes genes in extended MHC region. Chr., chromosome; n.s., not significant at transcriptome-wide significance threshold (P<2.2e-6). Light/dark banding is for visual clarity. The table includes all genes passing TWAS filters in any of the three tissues (i.e. significant TWAS p-value, significant model prediction p-value and no colocalization evidence of independent signals). Green shading in "Model pred. filter" and "Min. coloc PP3" columns indicate that the gene passed these filters for the given tissue. In the "Max. coloc PP4" column, green shading indicates further evidence of colocalized psoriasis/eQTL signal with posterior probability (PP) >0.8, and yellow shading with PP>0.5.

File Name: Supplementary Data 13
Description: TWAS results summarised by genomic region. TWAS, transcriptome-wide association study. Results are presented both before and after filtering transcriptome-wide significant TWAS associations using additional criteria based on model prediction p-value and coloc "PP3" evidence for independent signals (see Methods).

File Name: Supplementary Data 14
Description: Gene sets with strongest evidence for enrichment of genes in psoriasis-associated regions. Gene set enrichment assessed using DEPICT. GO, Gene Ontology; PPI, protein-protein interaction subnetwork; KEGG, Kyoto Encylopedia of Genes and Genomes.

File Name: Supplementary Data 15
Description: Functional enrichment of TWAS gene clusters. Top five enriched MSigDB genesets representing GO Biological Processes ("GOBP") or Human Phenotype Ontology terms ("HP") for each TWAS gene cluster. Ranked by: (1) overlap size, (2) hypergeometric test p-value (P_{hyper}, one-sided hypergeometric test).

File Name: Supplementary Data 16
Description: Enrichment between psoriasis TWAS genes and stimulated keratinocyte transcriptomes. DE, differentially expressed following cytokine stimulation.

File Name: Supplementary Data 17
Description: Genetic correlations with psoriasis susceptibility. Results of estimating genetic correlation (r_g) between psoriasis susceptibility and 592 other traits. Table is sorted by (i) correlation significant/not significant, (ii) correlation point estimate. Yellow and blue cells denote traits with significant positive and negative genetic correlation with psoriasis, respectively.

File Name: Supplementary Data 18
Description: Partial genetic causality between psoriasis and other traits. Results of estimating genetic causality proportion (GCP) for all 284 tested traits for which genetic correlation (r_g) is significantly non-zero (FDR<0.05). FDR, false discovery rate.

File Name: Supplementary Data 19
Description: Mendelian randomization analysis for significant LCV traits. Causal estimates derived from two-sample Mendelian randomization (Wald ratio/inverse-variance weighted test) for significant LCV traits. GCP: genetic causality proportion; CI: confidence interval; green highlighting: nominally significant p-value.

File Name: Supplementary Data 20
Description: Summary of psoriasis-associated regions, independent signals and implicated genes.

File Name: Supplementary Data 21
Description: Summary of previously reported non-European psoriasis loci. Includes most significant previously reported associations at loci outside of meta-analysis loci (LD blocks).

File Name: Supplementary Data 22
Description: Studies included in meta-analysis. N_{eff}, effective sample size; HUNT, The Trøndelag Health Study; KCL, King's College London; UCSF, University of California San Francisco; UM, University of Michigan.

File Name: Supplementary Data 23
Description: Details of ethics approval, ascertainment, genotyping, quality control, genome-wide imputation, and association testing for each contributing study.
