# Supplementary material for: GWAS meta-analysis of psoriasis identifies new susceptibility alleles impacting disease mechanisms and therapeutic targets
Source: Nat Commun. 2025 Feb 28;16:2051. doi: 10.1038/s41467-025-56719-8 (PMC11871359; doi:10.1038/s41467-025-56719-8)
Supplement: Supplementary file 4 — Reporting Summary [file 41467_2025_56719_MOESM4_ESM.pdf]

nature portfolio

Corresponding author(s): Michael A Simpson and James T Elder

Last updated by author(s): Dec 12, 2024

Reporting Summary

Nature Portfolio wishes to improve the reproducibility of the work that we publish. This form provides structure for consistency and transparency in reporting. For further information on Nature Portfolio policies, see our Editorial Policies and the Editorial Policy Checklist.

Statistics

For all statistical analyses, confirm that the following items are present in the figure legend, table legend, main text, or Methods section.

n/a	Confirmed	
☐	☒	The exact sample size (*n*) for each experimental group/condition, given as a discrete number and unit of measurement
☒	☐	A statement on whether measurements were taken from distinct samples or whether the same sample was measured repeatedly
☐	☒	The statistical test(s) used AND whether they are one- or two-sided<br>*Only common tests should be described solely by name; describe more complex techniques in the Methods section.*
☐	☒	A description of all covariates tested
☐	☒	A description of any assumptions or corrections, such as tests of normality and adjustment for multiple comparisons
☐	☒	A full description of the statistical parameters including central tendency (e.g. means) or other basic estimates (e.g. regression coefficient) AND variation (e.g. standard deviation) or associated estimates of uncertainty (e.g. confidence intervals)
☐	☒	For null hypothesis testing, the test statistic (e.g. *F*, *t*, *r*) with confidence intervals, effect sizes, degrees of freedom and *P* value noted<br>*Give P values as exact values whenever suitable.*
☐	☒	For Bayesian analysis, information on the choice of priors and Markov chain Monte Carlo settings
☒	☐	For hierarchical and complex designs, identification of the appropriate level for tests and full reporting of outcomes
☐	☒	Estimates of effect sizes (e.g. Cohen's *d*, Pearson's *r*), indicating how they were calculated

Our web collection on statistics for biologists contains articles on many of the points above.

Software and code

Policy information about availability of computer code

Data collection

Several of the studies that comprise the meta-analysis have not been described in previous publications, and took different approaches to genotype calling and quality control. These are described fully in Supplementary Data 23, and employed software including GenomeStudio v.2011.1 and Affymetrix Power Tools v 1.18.0 for genotype calling; PLINK v1.9, KING v1.4 and v1.9, BAFRegress (https://genome.sph.umich.edu/wiki/BAFRegress) and flashpca v2.0 for QC; and EAGLE v2.3, Minimac3, Beagle v.28Sep18.793, PBWT (https://github.com/richarddurbin/pbwt) and IMPUTE2 for phasing and genome-wide imputation.

Data analysis

Cross-study relatedness testing was performed using KING v2.0.
Association testing for each contributing study was performed using either PLINK v2.0, SNPTEST v2.5.2 or v2.5.4-beta3, or SAIGE v0.29.
Meta-analysis was performed using METAL v2020-05-05.
Post-processing was performed using GCTA-COJO, version 1.93.3-beta, and the validity of the LD reference panel was assessed using the R package susieR (v0.12.27).
LD Score regression and heritability estimation was performed using LDSC v1.0.1, and variance explained was estimated using the R package Mangrove (v1.21).
Annotation of coding variants was performed using the Variant Effect Predictor (http://grch37.ensembl.org/Homo_sapiens/Tools/VEP)
Regulatory predictions were generated using Regulome-TURF (https://github.com/Boyle-Lab/RegulomeDB-TURF).
Mediated expression analysis was performed using MESC (https://github.com/douglasyao/mesc).
TWAS, genetic correlation and partial genetic causality analyses were performed using pipelines provided as part of the Complex Trait Genetics Virtual Lab (https://vl.genoma.io/, analysed Jan 2022).
Gene positions were annotated using the biomaRt (v2.42.1) R package.
Colocalization analysis was performed using the coloc R package (v5.2.2).

Functional enrichment analysis was performed using DEPICT v1.1.
Mendelian Randomization was performed using the TwoSampleMR R package (v0.5.8).
Additional basic statistical analyses and tabulation/visualisation of data were performed using R software (version 3.6.3) including packages reshape2 (v.4.4), gridExtra (v2.3), ggrepel (v0.9.1), ggpubr (v0.5.0), ggplot2 (v3.4.0) and data.table (v1.12.8).

For manuscripts utilizing custom algorithms or software that are central to the research but not yet described in published literature, software must be made available to editors and reviewers. We strongly encourage code deposition in a community repository (e.g. GitHub). See the Nature Portfolio guidelines for submitting code & software for further information.

Data

Policy information about availability of data

All manuscripts must include a data availability statement. This statement should provide the following information, where applicable:

- Accession codes, unique identifiers, or web links for publicly available datasets
- A description of any restrictions on data availability
- For clinical datasets or third party data, please ensure that the statement adheres to our policy

The meta-analysis summary statistics generated in this study have been deposited in the GWAS Catalog under accession code GCST90472771 [https://ftp.ebi.ac.uk/pub/databases/gwas/summary_statistics/GCST90472001-GCST90473000/GCST90472771/].

This study used a custom LD reference panel comprising six GWAS datasets. Individual level genotype data for the CASP GWAS, PsA GWAS, and Exomechip case-control studies are available on dbGaP (dbGaP: phs000019.v1.p1 [https://www.ncbi.nlm.nih.gov/projects/gap/cgi-bin/study.cgi?study_id=phs000019.v1.p1], phs000982.v1.p1 [http://www.ncbi.nlm.nih.gov/projects/gap/cgi-bin/study.cgi?study_id=phs000982.v1.p1], and phs001306.v1.p1 [http://www.ncbi.nlm.nih.gov/projects/gap/cgi-bin/study.cgi?study_id=phs001306.v1.p1]), and WTCCC2 genotype data are archived at the European Genome-Phenome Archive (study ID EGAS00000000108 [https://ega-archive.org/studies/EGAS00000000108]). Data sharing restrictions do not allow making genotype data publicly available for the remaining two case-control cohorts. However, LD matrices based on the full reference panel for all 109 susceptibility loci have been deposited in the King's College London research data repository, KORDS, at https://doi.org/10.18742/27982057.

This study used publicly available reference data accessed through bioinformatics tools and provided by their developers: LD scores with LDSC (https://github.com/bulik/ldsc), variant annotations with VEP (https://grch37.ensembl.org/Homo_sapiens/Tools/VEP) and TURF (https://github.com/Boyle-Lab/RegulomeDB-TURF), expression scores with MESC (https://github.com/douglasyao/mesc/wiki/Download-expression-scores), gene annotations with DEPICT (https://github.com/perslab/depict).

This study used GTEX v7 eQTL data, accessed at https://gtexportal.org/home/downloads/adult-gtex/qtl.

This study used annotated gene sets from the Molecular Signatures Database v2023.2.Hs (https://www.gsea-msigdb.org/gsea/msigdb/).

The scRNA-seq data used in this study are deposited in the Gene Expression Omnibus under accession code GSE173706 [https://www.ncbi.nlm.nih.gov/geo/query/acc.cgi?acc=GSE173706], and the cytokine-stimulated keratinocyte expression data under accession code GSE255828 [https://www.ncbi.nlm.nih.gov/geo/query/acc.cgi?acc=GSE255828].

Genetic correlation and causal analysis were conducted for a wide range of traits using GWAS summary statistics compiled by the Complex Trait Genetics Virtual Lab (https://vl.genoma.io/). Further analyses were conducted using GWAS summary statistics made available by the Neale Lab (http://www.nealelab.is/uk-biobank/), the GLIDE consortium (https://data.bris.ac.uk/data/dataset/2j2rqgzedxlq02oqbb4vmycnc2), or deposited in GWAS Catalog under accession code GCST002216 [http://ftp.ebi.ac.uk/pub/databases/gwas/summary_statistics/GCST002001-GCST003000/GCST002216].

Research involving human participants, their data, or biological material

Policy information about studies with human participants or human data. See also policy information about sex, gender (identity/presentation), and sexual orientation and race, ethnicity and racism.

Reporting on sex and gender	Sex-stratified analyses were not conducted in this study.
Reporting on race, ethnicity, or other socially relevant groupings	This meta-analysis was based on GWAS analyses performed using participants of genetically homogeneous ancestry (after exclusion of outliers during standard predetermined QC processes), presumed to be European based on the geographic origin of the ascertained groups and in some cases through participants' self-reported ethnicity (e.g. UK Biobank: Data-Field 21000). We confirm that the final inclusion of participants was based only on having consistent genetic ancestry, so as to avoid introducing statistical bias due to population structure. None of our analyses included race, ethnicity or other socially-constructed grouping as a variable.
Population characteristics	This is a meta-analysis of several cohorts of participants from Canada, Estonia, Germany, Norway, UK and USA. Age, sex distribution and genotyping conditions were all varied, and in most cases not considered as covariates in our analyses. A full description of study cohorts are provided in Supplementary Data 23.
Recruitment	We provide detailed descriptions of the 18 studies included in the meta-analysis in Supplementary Data 23.
Ethics oversight	All studies were approved by appropriate institutional review boards.. Studies were conducted according to the Declaration of Helsinki principles, and participants provided informed consent. Ethics statements from each contributing study are provided in Supplementary Data 23.

Note that full information on the approval of the study protocol must also be provided in the manuscript.

Field-specific reporting

Please select the one below that is the best fit for your research. If you are not sure, read the appropriate sections before making your selection.

[x] Life sciences   [ ] Behavioural & social sciences   [ ] Ecological, evolutionary & environmental sciences

For a reference copy of the document with all sections, see nature.com/documents/nr-reporting-summary-flat.pdf

Life sciences study design

All studies must disclose on these points even when the disclosure is negative.

Sample size	The sample size was the largest that was available to the coordinating group at the time the study was convened, and represents the largest genetic study of psoriasis. It is widely accepted that robustly conducted GWAS meta-analyses that include tens of thousands of disease cases are well powered to detect biologically relevant genetic susceptibility loci.
Data exclusions	Within the 18 constituent studies, standard predetermined QC measures were employed to exclude samples due to low genotyping quality, relatedness or outlying ancestry (described in Supplementary Data 23). Following association testing and prior to the central meta-analysis, variants were excluded for individual studies if they had imputation quality score <0.7 or, where provided, minor allele frequency < 0.5%
Replication	Replication is an integral part of the meta-analysis model; we consider and report on heterogeneity in effect size estimates across 18 constituent studies. All genome-wide significant associations are considered to be replicated by consensus of included studies.
Randomization	Not applicable (observational study)
Blinding	Not applicable (observational study)

Reporting for specific materials, systems and methods

We require information from authors about some types of materials, experimental systems and methods used in many studies. Here, indicate whether each material, system or method listed is relevant to your study. If you are not sure if a list item applies to your research, read the appropriate section before selecting a response.

Materials & experimental systems

n/a	Involved in the study
[x]	[ ] Antibodies
[x]	[ ] Eukaryotic cell lines
[x]	[ ] Palaeontology and archaeology
[x]	[ ] Animals and other organisms
[x]	[ ] Clinical data
[x]	[ ] Dual use research of concern
[x]	[ ] Plants

Methods

n/a	Involved in the study
[x]	[ ] ChIP-seq
[x]	[ ] Flow cytometry
[x]	[ ] MRI-based neuroimaging

Plants

Seed stocks	*Report on the source of all seed stocks or other plant material used. If applicable, state the seed stock centre and catalogue number. If plant specimens were collected from the field, describe the collection location, date and sampling procedures.*
Novel plant genotypes	*Describe the methods by which all novel plant genotypes were produced. This includes those generated by transgenic approaches, gene editing, chemical/radiation-based mutagenesis and hybridization. For transgenic lines, describe the transformation method, the number of independent lines analyzed and the generation upon which experiments were performed. For gene-edited lines, describe the editor used, the endogenous sequence targeted for editing, the targeting guide RNA sequence (if applicable) and how the editor was applied.*
Authentication	*Describe any authentication procedures for each seed stock used or novel genotype generated. Describe any experiments used to assess the effect of a mutation and, where applicable, how potential secondary effects (e.g. second site T-DNA insertions, mosiacism, off-target gene editing) were examined.*
